# Sleep Modulates Alcohol Toxicity in *Drosophila*

**DOI:** 10.3390/ijms232012091

**Published:** 2022-10-11

**Authors:** Aliza K. De Nobrega, Eric J. Noakes, Natalie A. Storch, Alana P. Mellers, Lisa C. Lyons

**Affiliations:** 1 Program in Neuroscience, Department of Biological Science, Florida State University, Tallahassee, FL 32306, USA; 2 Department of Biological Sciences, University of Southern California, Los Angeles, CA 90007, USA

**Keywords:** *Drosophila*, sleep deprivation, alcohol, sedation, tolerance, toxicity, neural plasticity

## Abstract

Alcohol abuse is a significant public health problem. While considerable research has shown that alcohol use affects sleep, little is known about the role of sleep deprivation in alcohol toxicity. We investigated sleep as a factor modulating alcohol toxicity using *Drosophila melanogaster*, a model for studies of sleep, alcohol, and aging. Following 24 h of sleep deprivation using a paradigm that similarly affects males and females and induces rebound sleep, flies were given binge-like alcohol exposures. Sleep deprivation increased mortality, with no sex-dependent differences. Sleep deprivation also abolished functional tolerance measured at 24 h after the initial alcohol exposure, although there was no effect on alcohol absorbance or clearance. We investigated the effect of chronic sleep deprivation using mutants with decreased sleep, *insomniac* and *insulin-like peptide 2*, finding increased alcohol mortality. Furthermore, we investigated whether pharmacologically inducing sleep prior to alcohol exposure using the GABA_A_-receptor agonist 4,5,6,7-tetrahydroisoxazolo(5,4-c)pyridin-3-ol (THIP) mitigated the effects of alcohol toxicity on middle-aged flies, flies with environmentally disrupted circadian clocks, and flies with short sleep. Pharmacologically increasing sleep prior to alcohol exposure decreased alcohol-induced mortality. Thus, sleep prior to binge-like alcohol exposure affects alcohol-induced mortality, even in vulnerable groups such as aging flies and those with circadian dysfunction.

## 1. Introduction

Alcohol abuse and its associated pathologies are pervasive societal problems with serious negative impacts on individual health, family structure, and the economy [1,2,3,4,5,6,7,8]. In the United States, alcohol use disorders account for 79% of all diagnoses of substance use disorders [9], and the economic impact of alcohol misuse is estimated at USD 249 billion annually [7,10]. Alcohol abuse and alcohol pathologies appear higher in populations in which sleep deprivation is common, including teenagers, young adults, shift workers, and aged individuals [11,12,13,14,15,16,17,18]. Although considerable behavioral research has demonstrated the effects of alcohol on sleep homeostasis [19,20,21], surprisingly little is known about the role of sleep in modulating alcohol sensitivity and toxicity at the physiological level. Sleep impairments are traditionally viewed as symptoms of alcohol use disorders; however, sleep disorders increase the incidence and risk of relapse in recovering alcoholics [22,23,24,25]. Sleep deprivation represents a significant rising public health problem in the United States and the world [26,27,28,29]. The pervasiveness of factors contributing to sleep disruptions, including artificial light at night, the use of personal electronics, and the increase in shiftwork and extended work days [30,31], combined with the increased risk of substance abuse associated with sleep deprivation makes understanding how sleep deprivation affects alcohol-induced behaviors and toxicity imperative to identify and optimize therapies for the future prevention and treatment of alcohol-induced pathologies.

The high degree of physiological, molecular, and neurological conservation between the fruit fly *Drosophila melanogaster* and mammals makes *Drosophila* an ideal model for the investigation of sleep and alcohol interactions [32,33,34]. Sleep in *Drosophila* occurs in stages, varying in intensity during the night with observable sex- and age-dependent differences [35,36,37,38,39,40,41,42,43,44]. As in other species, circadian and homeostatic processes regulate sleep in flies, with waking activity affecting sleep need [38,45]. Moreover, alcohol physiology is remarkably conserved from flies to humans with parallels in behaviors, as well as the underlying molecular mechanisms [46,47]. When exposed to alcohol vapor, initially flies exhibit hyperactivity with increased locomotor activity, followed by a loss in motor control and, eventually, sedation [48,49,50,51]. Flies also develop functional alcohol tolerance dependent upon changes in neural plasticity [50,52,53,54,55] and addiction-like behaviors [56,57,58], with a preference for alcohol following previous exposure [59,60].

We investigated the role of sleep deprivation on alcohol responses using a group sleep deprivation protocol. Group sleep deprivation increases rebound sleep in male and female flies. We find that 24 h of acute sleep deprivation increase behavioral sensitivity and mortality following acute and repeated exposure to alcohol. Furthermore, the effects of sleep deprivation eliminate sex-based differences in alcohol responses and alcohol-induced mortality. Approximately 72 h of recovery sleep is necessary to return alcohol responses to baseline following 24 h of acute sleep deprivation. These effects are independent of alcohol metabolism, as no differences are observed in alcohol absorption and clearance between sleep deprived and non-sleep-deprived flies. Sleep deprivation also inhibits the induction of long-term functional alcohol tolerance, observed 24 h following the first alcohol exposure in both male and female flies, although short-term tolerance measured 4 h following the first alcohol exposure is not affected. Chronic sleep restriction using short-sleep phenotype genetic mutants also increases alcohol-induced mortality. Encouragingly, we find that pharmacologically increasing sleep has the opposite effect of sleep deprivation, ameliorating alcohol mortality in middle-aged flies and flies with disrupted circadian clocks. This research highlights the critical role of sleep as a factor in alcohol toxicity.

## 2. Results

### 2.1. Sleep Deprivation Increases Sensitivity to Alcohol-Induced Sedation

**Figure 1:**
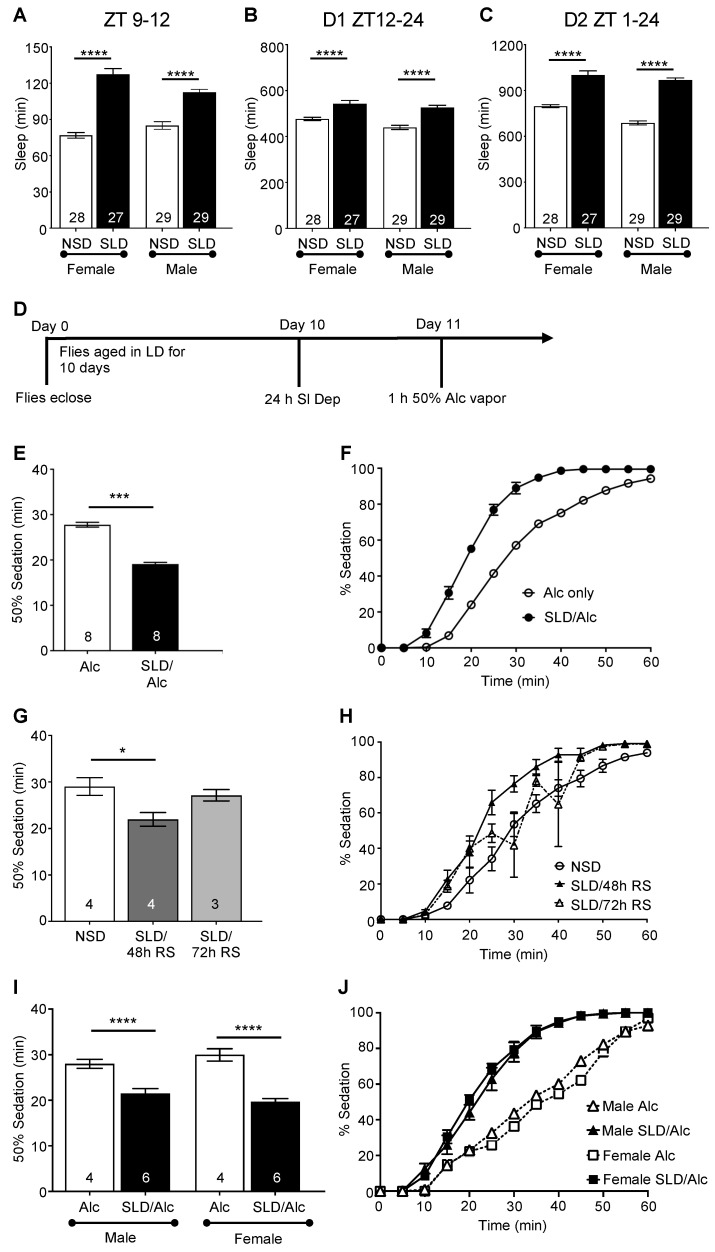
Acute sleep deprivation increases sensitivity to alcohol-induced sedation (**A**–**C**). Comparison of sleep profiles between 10 d wild-type sleep-deprived (SLD) and non-sleep-deprived (NSD) CS flies housed in LD cycles. Compared to NSD flies, both male and female SLD flies exhibited significantly increased quiescence immediately following the end of the sleep deprivation protocol ((**A**), ZT 9-12 [ANOVA: *F_3,108_* = 52.55, *p* < 0.0001]; * indicates significant differences between NSD and SLD male and female flies as calculated by Bonferroni post hoc analysis; * *p* < 0.05, *** *p* < 0.001, **** *p* < 0.0001), as well as during the 12 h dark cycle ((**B**), D1 ZT 12-24 ANOVA: *F_3,108_* = 21.16, *p* < 0.0001) and the second day ((**C**), D2 ZT 1-24 [ANOVA: *F_3,108_* = 76.85, *p* < 0.0001) after sleep deprivation, reflecting rebound sleep in SLD flies. (**D**) Wild-type CS 10 d old flies were sleep-deprived for 24 h and then exposed to 50% alcohol vapor for 1 h. Sensitivity to sedation was measured by counting the number of flies sedated every 5 min. (**E**) Sleep deprivation significantly exacerbated alcohol-induced sedation (*t*_(14)_ = 13.4558, *p* = 0.0002). Mean time necessary for 50% of the flies to become sedated during alcohol exposure and standard error of the mean are plotted. (**F**) Complete time course of alcohol exposure showing percent of flies exhibiting sedation for 10 d old sleep-deprived and non-sleep-deprived flies. (**G**,**H**) Effect of recovery sleep on alcohol-induced sedation. Wild-type CS flies were allowed to recover from sleep deprivation for either 48 h or 72 h prior to alcohol exposure, with behavioral sensitivity assessed. An amount of 48 h recovery sleep (RS) was insufficient to completely restore normal responses to alcohol-induced sedation in 10 d old SLD wild-type flies. (**G**) [ANOVA: *F_3,8_* = 24.6, *p* < 0.05]; * indicates significant differences between groups as calculated by Bonferroni post hoc analysis. An amount of 72 h recovery sleep restored normal behavioral sensitivity to alcohol sedation. (**H**) Complete time course of alcohol exposure showing percent of flies exhibiting sedation for 10 d old NSD flies and SLD flies with recovery sleep. (**I**) Separate groups of 10 d male and female flies were sleep-deprived for 24 h and then exposed to 50% alcohol vapor for 1 h (ANOVA *F_3,28_* = 29.24, *p* < 0.0001). N shown on bars for each group is the number of vials tested for each group, with 25–30 flies per vial. (**J**) Complete time course of alcohol exposure showing percent of flies exhibiting sedation for 10 d sleep-deprived and non-sleep deprived male and female flies.

Sleep deprivation appears to be a contributing factor to the increased use of alcohol, as suggested in studies of shift workers and young adults [61,62,63,64,65,66], although little is known about the effects of sleep loss on alcohol pathologies. In order to study the effect of sleep deprivation on alcohol sensitivity, it was necessary to develop a group-housed method of sleep deprivation. Flies in isolation with single housing exhibited a decreased number of synapses [67] and showed a reduced response to alcohol [68]. Furthermore, when males and females were separated and not allowed to mate, male flies showed increased alcohol preferences and changes in reward-signaling pathways [56]. Male and female sleep-deprived flies showed significant rebound sleep in the 3 h following sleep deprivation, as well as increased sleep during the night and the next day, compared to non-sleep-deprived flies (Figure 1A–C). Sleep-deprived flies using mechanical group sleep deprivation exhibited shorter sleep bouts with an increased number of sleep bouts, demonstrating sleep fragmentation for the first two days following sleep deprivation. The need for additional sleep was reduced by days 4 and 5, although small significant differences remained in the total sleep between sleep-deprived and non-sleep-deprived flies (Supplemental Figure S1). Thus, group-housed mechanical sleep deprivation was an effective and gentle method of sleep deprivation.

*Drosophila* (mixed sex, 10 d old) were sleep-deprived using mechanical sleep deprivation for 24 h (ZT 8–ZT 8) and then exposed to 50% alcohol vapor (1 h exposure; Figure 1D). We used approximately 10-day-old flies to facilitate the investigation of sleep deprivation on alcohol toxicity, as this age group of wild-type flies retains a robust circadian clock with circadian modulation of alcohol sensitivity and demonstrates measurable alcohol-induced mortality following a single binge-like alcohol exposure [69]. Sleep-deprived flies became sedated significantly faster than non-sleep-deprived age-matched controls, indicating that sleep loss increased behavioral sensitivity to alcohol (Figure 1E,F; *t*_(14)_ = 13.46, *p* = 0.0002). Potentially, sleep-deprived flies could enter a deep sleep state resembling sedation, so we tested whether 24 h of sleep deprivation in flies exposed only to water vapor for 1 h resulted in sedation. We found virtually no sedation in the absence of exposure to alcohol, with no sedation occurring in three independent experiments (total of 136 flies tested with 0 sedation). In a fourth experiment, 2 flies out of 48 exhibited a sedation-like state 55 min after the experiment started. Thus, sleep deprivation alone did not result in a sedation state. To verify that the effects of mechanical sleep deprivation on sedation occurred through sleep deprivation, we sleep-deprived flies and then let them recover for either 48 or 72 h prior to alcohol exposure. We found that, by 72 h after acute sleep deprivation, there were no differences in sedation between sleep-deprived and non-sleep-deprived flies, whereas flies with only 48 h of recovery time still showed a significant reduction in the time necessary for 50% of the flies to become sedated with binge-like alcohol exposure (Figure 1G,H).

Although alcohol has been previously shown to differentially affect the rates of sedation and recovery in males and females, we hypothesized that 24 h of acute sleep deprivation would eliminate sex-based differences in alcohol sensitivity. To test this prediction, after the flies had mated, male and female flies were separated. Males and females were sleep-deprived (9–11 d old) for 24 h and then exposed to 50% alcohol vapor for 1 h with sedation assessed. We found that sleep deprivation eliminated the sex-based differences in alcohol sensitivity, with male and female groups exhibiting similar sedation rates (Figure 1I,J).

Given that the majority of *Drosophila* sleep studies have been performed using younger flies, we wanted to verify whether the effects of sleep deprivation on alcohol responses were similar in younger flies. We found that 24 h of acute sleep deprivation significantly increased alcohol sensitivity in both male and female 5–6 d old flies, as shown by considerably the reduced times for 50% of the flies to reach sedation (Supplemental Figure S2A–C). No sex-specific differences were observed in the sensitivity of the flies to alcohol after sedation. We also performed experiments using 3 d old mixed-sex populations, finding that 24 h of sleep deprivation in young flies also increased alcohol sensitivity, with flies showing significantly reduced times for sedation (Supplemental Figure S2D–F). Thus, these experiments demonstrated that sleep deprivation of group-housed flies was effective for both increasing homeostatic sleep pressure and eliminating sex-based differences in alcohol responses.

### 2.2. Sleep Deprivation Increases Acute and Chronic Alcohol Toxicity

**Figure 2:**
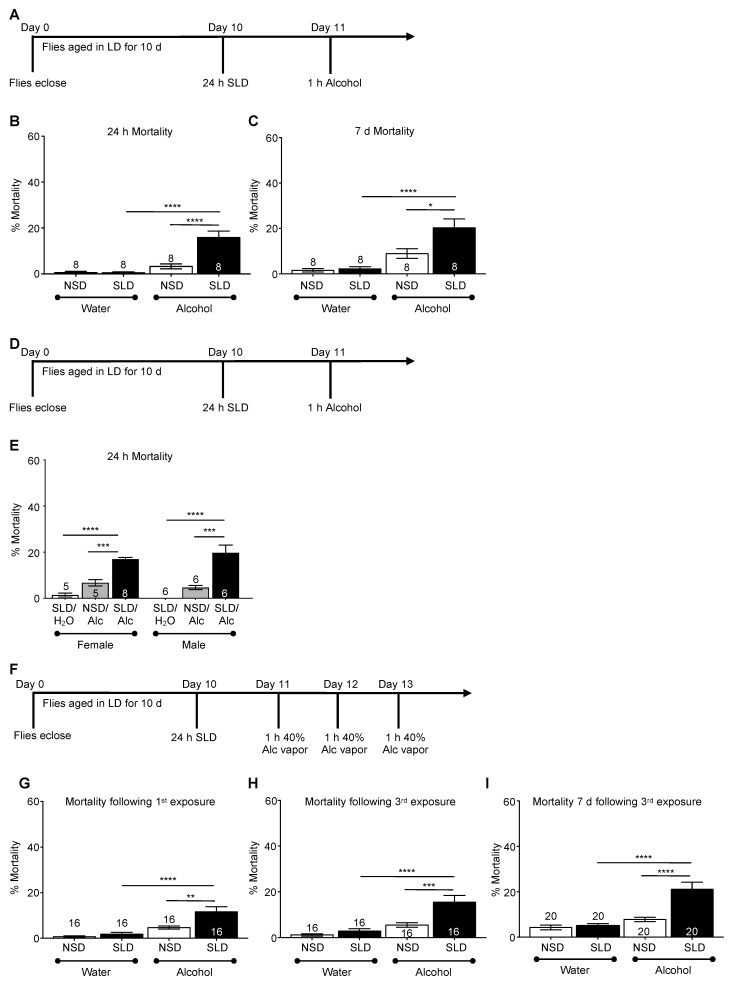
Acute sleep deprivation exacerbates alcohol-induced mortality following single or repeated exposure to alcohol. (**A**) Wild-type CS 10 d old flies were sleep deprived for 24 h and then exposed to 50% alcohol vapor for 1 h with mortality assessed every 24 h. (**B**) Sleep-deprived flies exhibited a significant increase in mortality within 24 h of exposure to alcohol compared to non-sleep-deprived flies (ANOVA *F*_3,28_ = 22.50, *p* < 0.0001). (**C**) Mortality differences remained apparent at 7 d after exposure to alcohol between non-sleep-deprived and sleep-deprived flies (ANOVA: *F_3,28_* = 14.01, *p* < 0.0001). (**D**,**E**) Separate groups of 10 d male and female flies were sleep-deprived for 24 h and then exposed to 50% alcohol vapor for 1 h. Sleep-deprived males and females exhibited significantly increased mortality 24 h following alcohol exposure (ANOVA *F*_5,32_ = 29.77, *p* < 0.0001), indicating no sex differences in the effects of acute sleep deprivation on alcohol-induced toxicity. (**F**) Wild-type CS 10 d old flies were sleep-deprived for 24 h, followed by 3 consecutive exposures to 1 h alcohol (50% alcohol vapor) at ZT 9 with each exposure separated by 24 h. (**G**) Sleep-deprived flies exhibited a drastic increase in mortality within 24 h of first exposure to alcohol compared to non-sleep-deprived flies (ANOVA: *F_3,76_* = 15.42, *p* < 0.0001). (**H**) Mortality continued to increase 24 h following the third exposure to alcohol vapor in both non-sleep-deprived and sleep-deprived flies (ANOVA: *F_3,76_* = 14.42, *p* < 0.0001). (**I**) Mortality measured at 7 d following the third alcohol exposure was significantly higher than mortality following the first alcohol exposure (ANOVA: *F_3,76_* = 19.91, *p* < 0.0001). * indicates significant differences between NSD and SLD groups as calculated by Bonferroni post hoc analysis; * *p* < 0.05, ** *p* < 0.01, *** *p* < 0.001, **** *p* < 0.0001.

Excessive binge drinking escalates the incidence of alcohol-poisoning deaths [70,71]. Therefore, it is important to understand the potential confounding effects of sleep loss on alcohol toxicity. We tested the effect of a single exposure to 50% alcohol vapor on mortality. Flies (10 d, mixed sex) were sleep-deprived for 24 h (ZT 8–ZT 8) and exposed to 50% alcohol vapor for one h at ZT 9 (Figure 2A). Nearly 100% of the flies became sedated under this protocol. Mortality was assessed at 24 h and 7 days following alcohol exposure. When exposed to alcohol vapor following sleep deprivation, flies showed significant mortality 24 h after alcohol exposure compared to non-sleep-deprived flies exposed to alcohol vapor or flies that were sleep-deprived and exposed to water vapor (Figure 2B, ANOVA: *F_3,28_* = 22.50, *p* < 0.0001 and Figure 2C; ANOVA: *F_3,28_* = 14.01, *p* < 0.0001). Non-sleep-deprived and sleep-deprived flies exposed to water vapor alone had negligible levels of mortality at either 24 h or 7 days, suggesting that 24 h of sleep deprivation itself did not result in mortal injury to the flies (Figure 2B,C). Exposure to alcohol vapor caused a noticeable but not significant rise in both immediate and delayed mortality compared to water vapor controls (Figure 2B,C). These results suggest that sleep deprivation exacerbated the acute toxicity of alcohol, with primary mortality observed within 24 h of alcohol exposure. To verify that the effects of sleep deprivation also eliminated sex-based differences in alcohol-induced mortality, separated male and female groups of flies were exposed to a single binge-like alcohol exposure (50% alcohol, vapor, 1 h) with mortality assessed. We found that mortality at 24 h after alcohol exposure was similar in sleep-deprived male and female groups (Figure 2E). In younger flies aged 5–6 d old, sleep deprivation also induced significant levels of mortality measured at 24 h after a single alcohol exposure (Supplemental Figure S3). We next investigated the effects sleep deprivation prior to a repeat binge alcohol exposure paradigm. As previously described, flies were sleep-deprived for 24 h (ZT 8–ZT 8) and then exposed to 40% alcohol vapor for 1 h (ZT 9) on three consecutive days (Figure 2F–I). Perhaps not surprisingly, the first alcohol exposure after sleep deprivation induced a significant increase in mortality (Figure 2G, ANOVA: *F_3,76_* = 15.42, *p* < 0.0001). Alcohol-induced mortality was not significantly higher following the second and third alcohol exposures (Figure 2H), potentially due to the opportunity for recovery sleep following the first exposure to alcohol. The degree of mortality observed at 7 days following the last alcohol exposure was similar between the acute and repeated binge alcohol paradigms (Figure 2I, ANOVA: *F_3,76_* = 19.91, *p* < 0.0001). Thus, sleep deprivation significantly increased alcohol toxicity in males and females, making acute sleep deprivation a potential risk factor for increased alcohol pathologies.

### 2.3. Sleep Deprivation Does Not Affect the Rate of Alcohol Clearance

**Figure 3:**
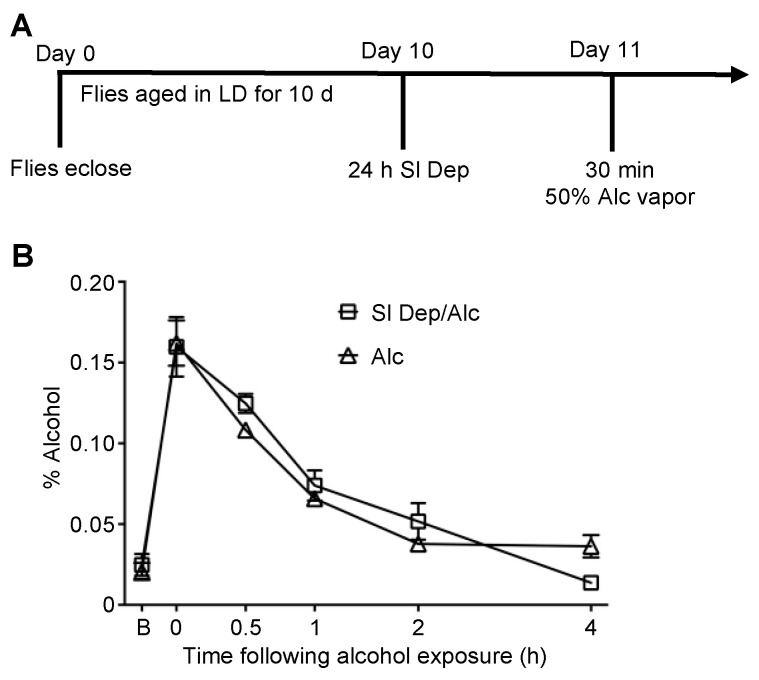
Sleep deprivation does not affect alcohol accumulation or rate of alcohol clearance. (**A**) Flies were aged in 12:12 LD cycle for 10 days and then sleep-deprived for 24 h. CS 10 d old flies were exposed to 30 min of 50% alcohol vapor on day 11 with alcohol absorbance and rate of alcohol clearance assessed. (**B**) No significant differences existed in alcohol absorbance or rate of alcohol clearance between sleep-deprived and non-sleep-deprived flies (*n* = 4 per group).

Potentially, the increases in alcohol sensitivity and mortality observed following sleep deprivation were due to increased alcohol absorption or a decline in the rate of alcohol clearance, resulting in greater alcohol exposure and subsequent toxicity. To investigate this possibility, flies were sleep-deprived as previously described and exposed to 50% alcohol vapor for 30 min at ZT 9, and alcohol absorbance was measured (Figure 3A). There were no significant differences in alcohol absorbance or clearance between sleep-deprived flies and non-sleep-deprived flies (Figure 3B). These results suggest that potential metabolic changes due to sleep deprivation did not account for the observed increased sensitivity to alcohol, with the more likely possibilities including sleep-deprivation-induced changes in neuroadaptation at the molecular or cellular levels.

### 2.4. Chronic Sleep Deprivation Induces Increased Alcohol-Induced Mortality

**Figure 4:**
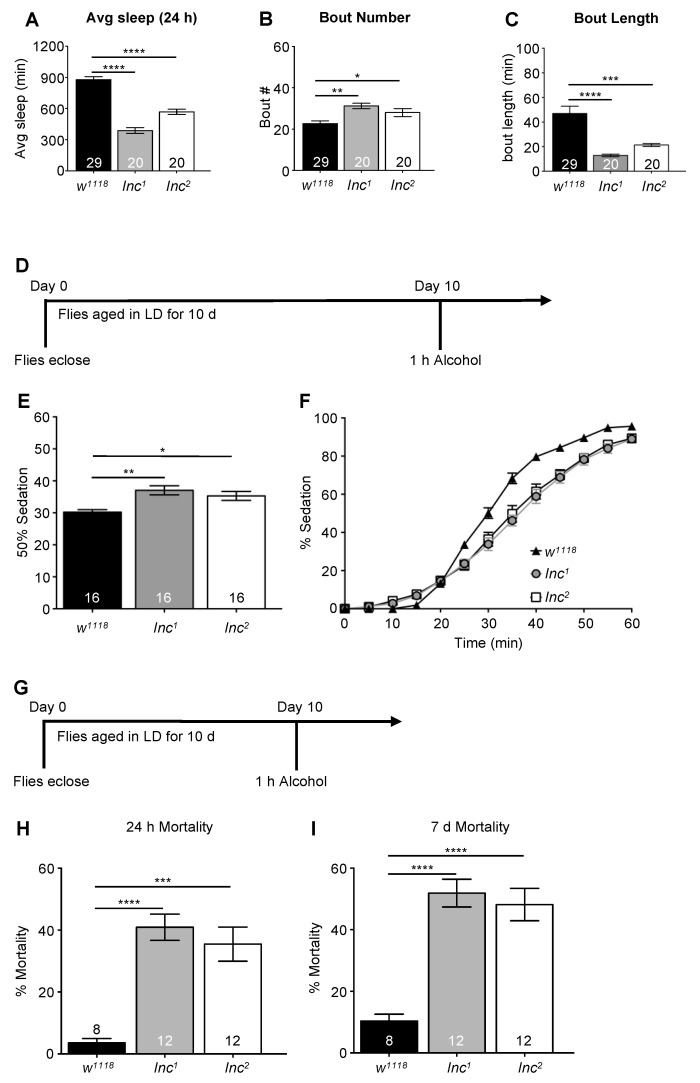
Mutations in *insomniac* significantly increase mortality following a single exposure to alcohol without increasing sensitivity to alcohol-induced sedation. The impact of sleep loss due to mutations in the *inc^1^* and *inc^2^* genes on alcohol sensitivity was assessed. (**A**–**C**) Sleep profiles of *w^1118^*, *inc^1^*, and *inc^2^* flies. *inc^1^* and *inc^2^* flies had significantly shorter daily sleep times ((**A**), ANOVA: *F_2,67_* = 81.13, *p* < 0.0001), increased number of bouts ((**B**), ANOVA: *F_2,67_* = 8.64, *p* < 0.0001), and shorter bout lengths ((**C**), ANOVA: *F_2,67_* = 17.52, *p* < 0.0001) compared to *w^1118^* flies. (**D**) *inc^1^*, *inc^2^*, and *w^1118^* control flies 10 d of age were exposed to 50% alcohol vapor for 1 h at ZT 9 with sedation assessed every 5 min during alcohol exposure. (**E**) *inc^1^* and *inc^2^* flies demonstrated increased resistance to alcohol vapor compared to *w^1118^* controls (ANOVA: *F_2,34_* = 47.28, *p* < 0.0001). (**F**) The complete time course for *w^1118^*, *inc^1^*, and *inc^2^* flies. (**G**–**I**) The impact of sleep loss due to mutations in *inc^1^* and *inc^2^* on alcohol-induced mortality was assessed. (**G**) *w^1118^*, *inc^1^*, and *inc^2^* 10 d old flies were exposed to 50% alcohol vapor for 1 h at ZT 9 with mortality assessed every 24 h for 7 d following exposure to alcohol. (**H**) *inc^1^* and *inc^2^* mutant flies showed significantly increased mortality 24 h following exposure to alcohol compared to *w^1118^* control flies (ANOVA: *F_2,29_* = 16.46, *p* < 0.0001). (**I**) Mortality in *inc^1^* and *inc^2^* flies continued to rise 7 d following the initial exposure to alcohol (ANOVA: *F_2,29_* = 20.87, *p* < 0.0001). * indicates significant differences between groups as calculated by Bonferroni post hoc analysis; * *p* < 0.05, ** *p* < 0.01, *** *p* < 0.001, **** *p* < 0.0001.

Chronic sleep deprivation comprised of multiple short-sleep nights may be a predisposing factor for increased alcohol consumption and other recreational drug use [18,72]. In the United States, approximately 70 million Americans suffer from chronic sleep loss with serious consequences for health and longevity, as well as economic productivity [29,73,74]. To investigate the effects of chronic sleep restriction on alcohol neurobiology, we employed a genetic approach rather than a mechanical system to induce sleep deprivation to avoid the possibility of stress arising from long-term mechanical stimulation. Numerous mutants with short-sleep phenotypes have been identified in *Drosophila*. However, the circadian clock also regulates aspects of sleep and sleep timing, and many sleep mutants have circadian phenotypes. Given previous research demonstrating circadian modulation of alcohol sensitivity and increased alcohol-induced mortality with circadian disruption [48,69], we used the mutant *insomniac* that has normal circadian rhythms but exhibits a short-sleep phenotype [75] to investigate the effects of chronic sleep restriction on alcohol toxicity. *Insomniac* (*inc*) is a mutation in a putative adaptor protein for the *Cullin-3* ubiquitin ligase complex [75]. *inc^1^* and *inc^2^* mutant lines have a 90% reduction in *inc* transcript mRNA levels, with no detectable protein produced [75]. Confirming previously published results, we found that that *inc^1^* and *inc^2^* male flies (Figure 4A–C) exhibited considerable reductions in total sleep time, with *inc^1^* flies sleeping a little over 300 min per day and *inc^2^* flies sleeping approximately 600 min per day (Figure 4A, ANOVA: *F_2,67_* = 81.13, *p* < 0.0001). These mutants exhibited significantly shortened sleep bouts (Figure 4B, ANOVA: *F_2,67_* = 17.52, *p* < 0.0001), reflecting a decrease in sleep consolidation, although they did have a greater number of sleep bouts (Figure 4C, ANOVA: *F_2,67_* = 8.64, *p* < 0.0001).

We exposed 10 d old *inc^1^* and *inc^2^* flies to 50% alcohol for 1 h at ZT 9 with sedation assessed at 5 min intervals (Figure 4D). Surprisingly, *inc^1^* and *inc^2^* flies did not exhibit increased sensitivity to alcohol; indeed, these mutants were more resistant to the sedating effects of alcohol, with significantly longer times to reach 50% sedation than the *w^1118^* control flies (Figure 4D,E, ANOVA: *F_2,34_* = 47.28, *p* < 0.0001, with post hoc analysis identifying significant differences between *w^1118^* vs. *inc^1^* and *w^1118^* vs. *inc^2^*). These results suggest that either compensatory mechanisms existed to buffer against increased sensitivity to alcohol in these mutants or chronic sleep loss associated with the disruption in the *Cullin-3* ubiquitin ligase complex did not increase alcohol sensitivity.

While the chronic sleep deficit associated with the disruption in the *Cullin-3* ubiquitin ligase complex in the *inc* mutants did not increase alcohol sensitivity, we hypothesized that it would still increase alcohol toxicity, as alcohol affects multiple signaling pathways, both in the central nervous system and in peripheral tissues. To test this, we gave 10 d *inc^1^* and *inc^2^* flies a single exposure to 50% alcohol vapor for 1 h at ZT 9 and assessed mortality at 24 h and 7 d following the alcohol exposure (Figure 4G). Both *inc^1^* and *inc^2^* flies exhibited significantly higher mortality immediately (24 h), as well as 7 d following the exposure, compared to *w^1118^* background controls (Figure 4H,I, ANOVA: *F_2,29_* = 16.46, *p* < 0.0001). Given that the *inc* mutants are postulated to have defects in ubiquitination that may affect many target proteins and signaling pathways, it is possible that the observed mortality was due to other consequences of the mutation and not to the effects of chronic sleep restriction on alcohol toxicity.

**Figure 5:**
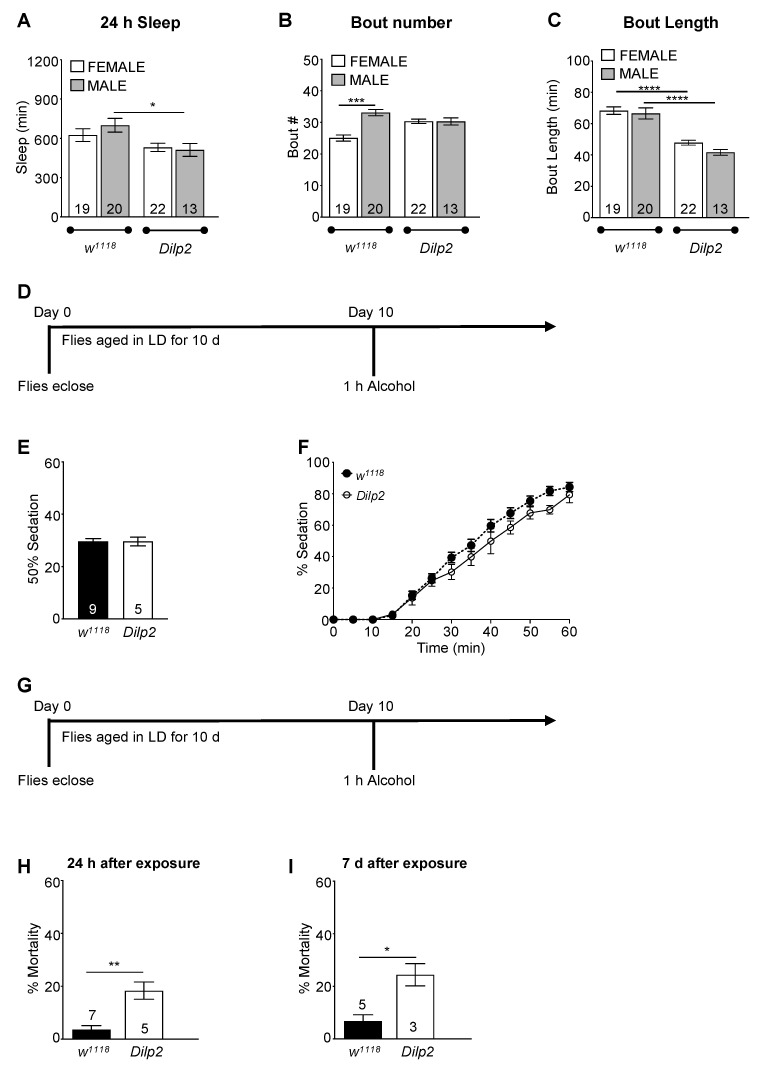
Short-sleeping *Dilp2* mutants have significantly increased mortality following a single exposure to alcohol with no effect on alcohol-induced sedation. (**A**–**C**) Sleep profiles of *w^1118^* and *Dilp2* flies. *Dilp2* male and female flies had significantly shorter daily sleep times ((**A**), ANOVA: *F_3,82_* = 3.646, *p* = 0.0160) and shorter bout lengths compared to *w^1118^* flies ((**C**), [ANOVA: *F_3,82_* = 25.57, *p* < 0.001], * indicates significant differences as calculated by Bonferroni post hoc analysis; (**B**) [ANOVA: *F_3,82_* = 13.81, *p* < 0.001], * indicates significant differences as identified by Bonferroni post hoc analysis). (**D**) *Dilp2* and *w^1118^* control flies 10 days of age were exposed to 50% alcohol vapor for 1 h at ZT 9 with sedation assessed every 5 min during alcohol exposure. (**E**) No significant differences in behavioral sensitivity for sedation were observed between *Dilp2* and w*^1118^* controls (*t*_(12)_ = 0.035, *p* = 0.9726). (**F**) The complete time course for *w^1118^* and *Dilp2* flies. (**G**–**I**) The impact of sleep loss due to mutations in *Dilp2* on alcohol-induced mortality was assessed. *w^1118^* and *Dilp2* 10 d flies were exposed to 50% alcohol vapor for 1 h at ZT 9 with mortality assessed every 24 h for 7 d following exposure to alcohol (**G**). *Dilp2* mutant flies showed significantly increased mortality 24 h following exposure to alcohol compared to *w^1118^* control flies (*t*_(10)_ = 4.567, *p* = 0.0010). (**H**) Mortality in *Dilp2* flies continued to rise 7 d following the initial exposure to alcohol (*t*_(6)_ = 3.974, *p* = 0.0073). * indicates significant differences between groups as calculated by Bonferroni post hoc analysis; * *p* < 0.05, ** *p* < 0.01, *** *p* < 0.001, **** *p* < 0.0001.

As the interference with the ubiquitin ligase complex in the *inc* mutants could affect other pathways influencing alcohol sensitivity, we wanted to confirm these results with another genetic mutant that exhibits a reduced sleep phenotype but a normal circadian clock. Insulin-like peptide 2 (*ilp2*) is one of seven insulin-like peptides present in *Drosophila*, although there is only one insulin-like receptor identified [76,77]. Although there are spatial and temporal differences in expression between the *Dilp* genes, there appears to be a high degree of compensation and functional redundancy, such that knockdown of an individual *Dilp* does not significantly affect insulin- or igf-signaling pathways [77]. Dilp2 mutants exhibit reduced sleep, although the function of the circadian system appears to be relatively preserved [76], making *Dilp2* a good candidate to test the effects of reduced sleep on alcohol toxicity. We verified that *Dilp2* mutant flies (male and female) exhibited a reduced sleep phenotype compared to *w^1118^* background controls with significantly shorter bout lengths, although the total bout numbers were similar (Figure 5A–C). The majority of *Dilp2* flies demonstrated circadian locomotor activity, with 69.3% of the mutant flies (*n* = 36) exhibiting circadian rhythms with a period length of 23.51 ± 0.08 h compared with *w^1118^* control flies (*n* = 61), in which 93% were rhythmic with a period length of 23.75 ± 0.04 h. We exposed 9-10 d old *Dilp2* flies to 50% alcohol for 1 h at ZT 9 with sedation assessed at 5 min intervals (Figure 5D). Similar to *inc* mutants, *Dilp2* flies did not show a greater sensitivity to alcohol, with similar sedation rates observed compared to *w^1118^* control flies (Figure 5E,F). However, *Dilp2* mutants had significantly higher levels of mortality at 24 h and 7 days after a single binge-like alcohol exposure compared to age-matched *w^1118^* control flies (Figure 5G–I). Together, the results from these studies indicate that separate mechanisms mediated behavioral sensitivity to alcohol and the alcohol’s toxic effects, whereby chronic sleep loss increased alcohol-induced mortality, but did not affect alcohol-induced sedation, unlike acute sleep deprivation.

### 2.5. Pharmacologically Increasing Sleep Ameliorates Alcohol-Induced Mortality in Populations with Sleep Phenotypes

**Figure 6:**
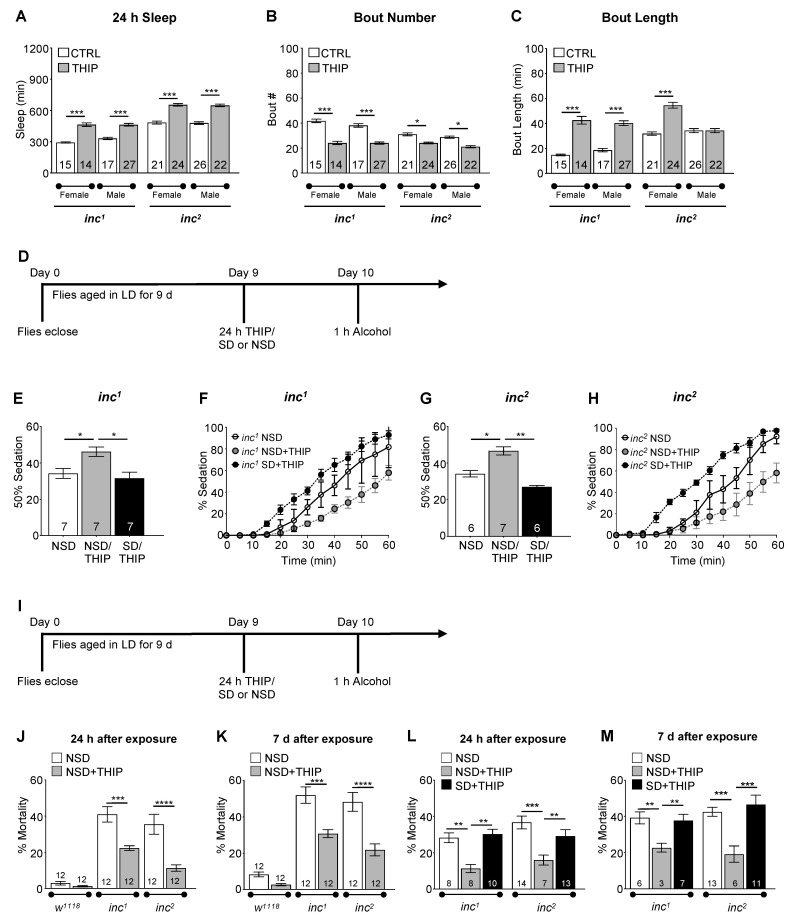
Pharmacologically increasing sleep in *insomniac* mutants significantly increases behavioral resistance to alcohol sedation and ameliorates alcohol-induced mortality. (**A**–**C**) Comparison of sleep profiles between *w^1118^* and THIP-fed *inc^1^* and *inc^2^* flies housed in LD cycles. Compared to control *inc^1^* and *inc^2^* flies, THIP-fed flies demonstrated significantly increased and consolidated sleep ((**A**), ANOVA: *F_7,158_*= 94.36, *p* < 0.001; * specifies significant differences between groups as indicated by Bonferroni post hoc analyses) with a decreased number of sleep bouts ((**B**), ANOVA: *F_7,158_* = 45.10, *p* < 0.001), as well as increased bout duration for most groups ((**C**), ANOVA: *F F_7,158_* = 42.62, *p* < 0.0001). (**D**–**H**) To determine whether THIP treatment and increased sleep buffered alcohol sensitivity, *inc^1^* and *inc^2^* flies were aged in LD cycles for 9 d and transferred to media containing 0.1 mg/mL THIP or received THIP treatment while being sleep-deprived for 24 h. On day 10, *inc^1^* and *inc^2^* flies were exposed to 50% alcohol vapor for 1 h at ZT 9 with sedation assessed every 5 min during alcohol exposure (**D**). Pharmacologically increasing sleep (NSD/THIP) in *inc^1^* and *inc^2^* flies significantly increased the resistance to alcohol-induced sedation compared to non-sleep-deprived (NSD) *inc^1^* and *inc^2^* flies ((**E**), ANOVA: *F_3,18_* = 12.22, *p* < 0.001; and (**G**), ANOVA: *F_3, 16_* = 44.38, *p* < 0.001, respectively; * specifies significant differences between groups). THIP treatment had no effect on alcohol sensitivity in sleep-deprived (SLD) *inc^1^* and *inc^2^* flies, indicating that the alcohol resistance observed in NSD THIP-treated flies was not due to buildup of GABA-A receptor tolerance. (**F**,**H**) The complete alcohol sedation time course. (**I**–**K**) To determine whether increased sleep buffered against the toxic effects of alcohol, *inc^1^*, *inc^2^*, and *w^1118^* flies were aged in LD cycles for 9 d and transferred to media containing 0.1 mg/mL THIP. On day 10, flies were exposed to 1 h of 50% alcohol vapor at ZT 9 with mortality assessed every 24 h for 7 d following exposure. Significantly reduced mortality was observed in THIP-fed *inc^1^* and *inc^2^* flies 24 h following alcohol exposure compared to *inc^1^* and *inc^2^* flies given alcohol alone ((**I**), ANOVA: *F_3,44_* = 13.27, *p* < 0.0001); * indicates significant differences between groups as calculated by Bonferroni post hoc analysis). (**J**) Although mortality rose in THIP-fed *inc^1^* and *inc^2^* flies 7 d following alcohol exposure, the percent of THIP-fed *inc^1^* and *inc^2^* flies was still significantly lower than non-THIP fed *inc^1^* and *inc^2^* flies at 7 d after exposure to alcohol (ANOVA: *F_2,44_* = 12.83, *p* < 0.0001). (**L**,**M**) To determine whether the decreased mortality observed in THIP-fed flies was due to GABA-A receptor activation or increased sleep, *inc^1^* and *inc^2^* flies were aged in LD cycles for 10 d and transferred to media containing 0.1 mg/mL THIP while being sleep-deprived for 24 h. At ZT 9 on day 10, the flies were exposed to 1 h of 50% alcohol vapor with mortality assessed every 24 h for 7 d following exposure. *inc^1^* and *inc^2^* flies sleep-deprived while on THIP media were significantly more susceptible to alcohol-induced mortality at 24 h (**L**) and 7 days (**M**) compared to non-sleep-deprived THIP-fed flies ((**L**), ANOVA: *F_5,54_* = 7.743, *p* < 0.001; (**M**), ANOVA: *F_5,40_* = 5.140, *p* < 0.001) * specifies significant differences between groups as calculated by Bonferroni post hoc analysis; * *p* < 0.05, ** *p* < 0.01, *** *p* < 0.001, **** *p* < 0.0001.

**Figure 7:**
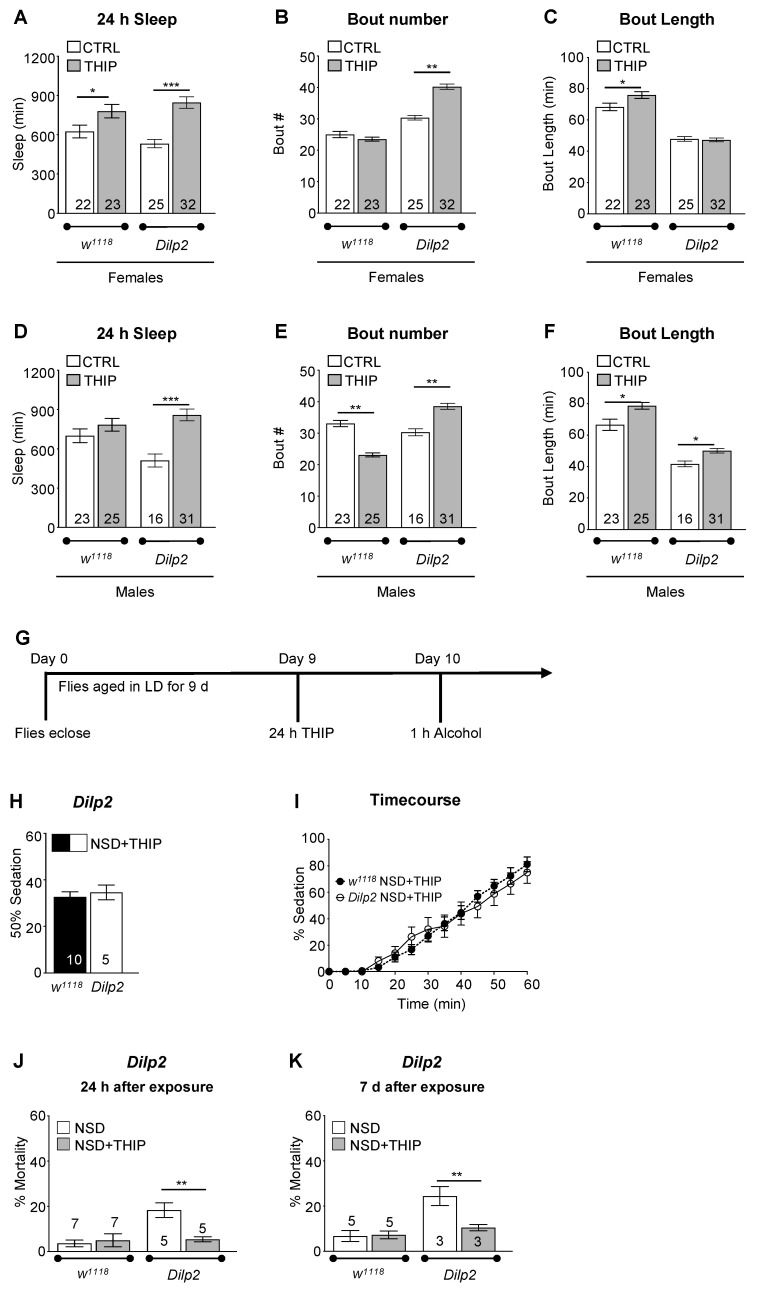
Pharmacologically increasing sleep in short-sleeping *Dilp2* mutants significantly reduces alcohol-induced mortality. (**A**–**F**) Comparison of sleep profiles between *w^1118^* and THIP-fed *w^1118^* and *Dilp2* flies housed in LD cycles. Compared to non-THIP-fed female *w^1118^* and *Dilp2* flies, THIP-fed flies demonstrated significantly increased total sleep ((**A**) [ANOVA: *F_3,98_* = 10.95, *p* < 0.001]; * specifies significant differences between groups as indicated by Bonferroni post hoc analyses). *Dilp2* THIP-fed females had significantly higher number of sleep bouts compared to non-THIP-fed controls ((**B**) ANOVA: *F_3,98_* = 98.62, *p* < 0.001), while THIP treatment had no effect on bout duration ((**C**) ANOVA: *F_3,98_* = 66.15, *p* < 0.001). *w^1118^* female flies treated with THIP showed increased bout length. Compared to non-THIP-fed male *Dilp2* flies, male THIP-treated flies had significantly increased sleep ((**D**) ANOVA: *F_3,91_* = 7.815, *p* < 0.001), with an increased number of sleep bouts ((**E**) ANOVA: *F_3,91_* = 55.54, *p* < 0.001) and slightly increased sleep duration ((**F**) ANOVA: *F_3,91_* = 46.36, *p* < 0.001). Male THIP-fed *w^1118^* flies exhibited significantly more consolidated sleep, as seen by decreased sleep bouts (**E**) and increased sleep bout duration (**F**), with no changes in total sleep amount (**D**). (**G**–**I**) To determine whether increased sleep buffered against the toxic effects of alcohol, flies were aged in LD cycles for 9 d and transferred to media containing 0.1 mg/mL THIP. Pharmacologically increasing sleep (NSD/THIP) in *Dilp2* mutants showed similar rates of sedation compared to *w^1118^* THIP-treated flies (*t_(13_*_)_ = 0.499, *p* = 0.6264). (**J**,**K**) Significantly reduced mortality was observed in THIP-treated *Dilp2* flies 24 h following alcohol exposure compared to non-THIP-treated flies alone ((**J**) ANOVA: *F_3,21_* = 8.106, *p* < 0.001) and 7 days later ((**K**) ANOVA: *F_3,13_* = 10.03, *p* < 0.01). * indicates significant differences between groups as calculated by Bonferroni post hoc analysis; * *p* < 0.05, ** *p* < 0.01, *** *p* < 0.001.

Previously we found that circadian arrhythmia and aging significantly increased alcohol-induced mortality [69], mirroring issues found in human populations such as shift-workers and elderly people with sleep disturbances. If accrued sleep loss was the driving force for the observed alcohol-induced mortality in *inc* flies and *Dilp2* mutants, we hypothesized that increasing sleep in these mutants should decrease mortality following exposure to alcohol. To pharmacologically increase sleep, *inc^1^* and *inc^2^* mutant flies were raised on standard *Drosophila* media for 9 d and then transferred to media containing the GABA_A_ agonist THIP (also known as Gaboxadol), which has previously been shown to pharmacologically increase sleep in *Drosophila* [78,79,80]. THIP treatment significantly increased sleep in *inc^1^* and *inc^2^* male and female flies (Figure 6A). THIP treatment also resulted in more consolidated sleep, as seen in a smaller number of sleep bouts with an increased length of sleep in each bout (Figure 6B,C). Similarly, THIP treatment significantly increased sleep in *w^1118^* control flies (Figure 7). In *w^1118^* flies, THIP resulted in significantly fewer but longer sleep bouts, indicating more consolidated sleep (Figure 7). Flies with THIP-induced increased sleep demonstrated reduced sensitivity to alcohol compared to non-THIP-treated flies or groups in which THIP-treated flies were simultaneously subjected to mechanical sleep deprivation (Figure 6D–H). THIP pre-treatment significantly reduced mortality at 24 h and 7 d following alcohol exposure in both *inc^1^* and *inc^2^* mutant flies compared to non-THIP-exposed *inc^1^* and *inc^2^* mutants (Figure 6I,J, ANOVA: *F_3,44_* = 13.27, *p* < 0.0001 and Figure 6K, ANOVA: *F_2,44_* = 12.83, *p* < 0.0001, respectively). However, THIP has dual effects as an analgesic and anxiolytic and has been tested as a treatment for alcohol use disorders, as well as insomnia [81]. Potentially, as an agonist for GABA_A_ receptors, THIP may affect alcohol–receptor interactions to affect mortality rather than through its pharmacological induction of sleep. To determine whether THIP interactions decreased alcohol-induced mortality by altering alcohol–receptor interactions rather than through increased sleep prior to alcohol exposure, we combined mechanical sleep deprivation with 24 h of THIP exposure and then assessed the response to a single binge-like alcohol exposure. Sleep-deprived *inc^1^* and *inc^2^* mutant flies on THIP media showed significantly increased alcohol-induced mortality when compared to *inc^1^* and *inc^2^* mutant flies on control media, although THIP treatment alone significantly decreased alcohol-induced mortality (Figure 6L,M). Thus, the mitigation of alcohol-induced mortality with THIP observed in the *inc* mutants appeared to occur through benefits arising from increased sleep.

Although *Dilp2* mutants exhibited reduced sleep, the mutation affected different signaling pathways than the *inc* mutation. We found that, similar to *inc* mutants and *w^1118^* control flies, THIP increased the total sleep in *Dilp2* male and female flies (Figure 7A–C, females; Figure 7D–F, males; non-THIP-treated *Dilp2* male and female data from Figure 5). THIP-treated *Dilp2* and *w^1118^* control flies exhibited similar rates of alcohol-induced sedation (Figure 7G–I). Flies with 24 h of THIP pre-treatment also showed a significant reduction in alcohol-induced mortality measured at 24 h and 7 d after a single alcohol exposure compared with non-THIP-treated flies (Figure 7J,K). These results were consistent with the hypothesis that increased sleep prior to binge-like alcohol exposure buffered the toxic effects of alcohol.

### 2.6. Pharmacologically Increasing Sleep Independent of Circadian Rhythmicity Decreases Alcohol-Induced Mortality

**Figure 8:**
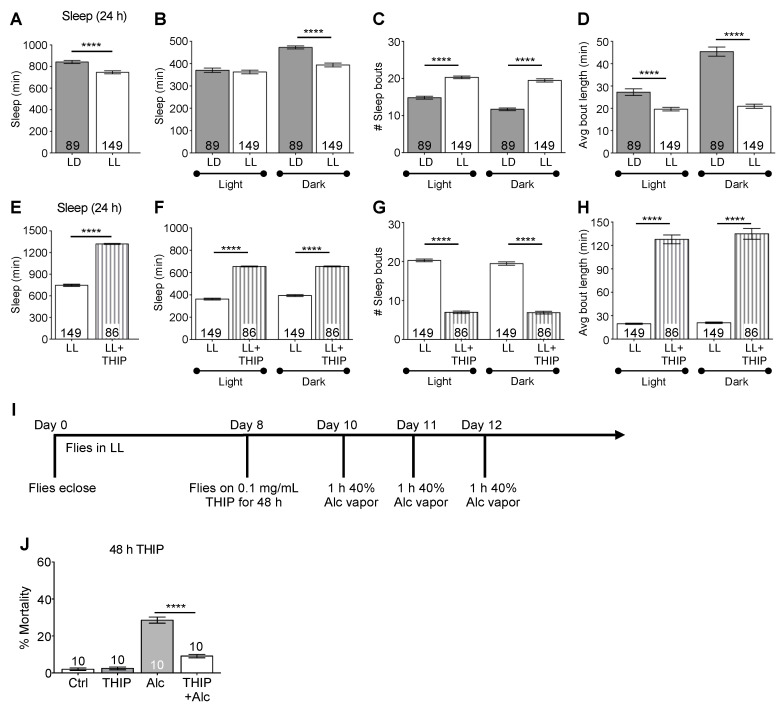
Pharmacologically increasing sleep in flies that are circadianly disrupted reduces mortality following repeated exposures to alcohol. (**A**–**D**) Comparison of sleep profiles between male 10 d CS flies housed in LD cycles and 10 d flies housed in constant light conditions (LL). Compared to control flies grown in a 12:12 h LD cycle, 10 d CS flies grown in LL exhibited significantly decreased total sleep time per day ((**A**) [*t*_(236)_ = 4.46, *p* < 0.0001)]) with decreased total sleep during the subjective night ((**B**) ANOVA: *F_3,442_* = 27.31, *p* < 0.0001). More fragmented sleep was observed in LL during the day and subjective night with an increased number of sleep bouts ((**C**) ANOVA: *F_3,442_* = 98.99, *p* < 0.0001) and decreased bout duration compared to flies maintained in LD ((**D**) ANOVA: *F_3,442_* = 78.45, *p* < 0.0001). LL 10 day flies fed 0.1 mg/mL THIP exhibited significantly more sleep compared to 10 d LL flies on control media ((**E**) *t*_(233)_ = 29.43, *p* < 0.0001), increased sleep time during both the day and subjective night ((**F**) ANOVA: *F_3,463_* = 437.1, *p* < 0.0001), decreased number of sleep bouts ((**G**) ANOVA: *F_3,463_* = 358.1, *p* < 0.0001), and increased bout duration ((**H**) ANOVA: *F_3,463_* = 277.6, *p* < 0.0001). (**I**) To determine whether increasing sleep was sufficient to ameliorate alcohol-induced mortality under conditions of circadian disruption, CS flies were housed in LL upon eclosion and transferred to media containing 0.1 mg/mL THIP on day 8 for 48 h. On days 10, 11, and 12, flies were subjected to a three-exposure repeated binge-like alcohol paradigm with 1 h alcohol exposure (50% alcohol vapor) occurring at ZT 9, and mortality was assessed. (**J**) Alcohol-induced mortality in THIP-fed LL flies was drastically reduced compared to control LL flies (ANOVA: *F_3,36_* = 132.6, *p* < 0.0001). * indicates significant differences between groups as calculated by Bonferroni post hoc analysis; **** *p* < 0.0001.

In *Drosophila*, the circadian clock can be rendered non-functional using environmental manipulation by housing the flies in constant light. Constant light (LL) is sufficient to dampen molecular oscillations and abolish circadian rhythms in locomotor activity, memory formation, and the rhythm in alcohol-induced loss-of-righting reflex [51,82,83,84,85,86,87]. We have previously shown that environmental disruption of circadian function exacerbates alcohol sensitivity and mortality [48,69]. Previously, we had hypothesized that the increased alcohol mortality observed in flies in LL was due to the disruption in the circadian clock. However, recent research on the effects of constant light has shown that, when very young flies starting at day 1 are maintained in constant light, flies demonstrate significantly increased sleep with longer sleep bout duration during the day and less sleep at night, presumably due to circadian dysfunction altering the timing of sleep. (Rodrigues et al. Free Radical Biology in the paper the reviewer mentioned). Along with a disrupted circadian clock, we found that 10 d CS flies in LL had significantly lower total sleep and, specifically, less sleep during the subjective night compared to 10 d CS flies in LD (Figure 8A, *t*_(236)_ = 4.46, *p* < 0.0001; Figure 8B, ANOVA: *F_3,442_* = 27.31, *p* < 0.0001), consistent with mis-timed sleep due to circadian dysfunction. Flies housed in LL had a significantly higher number of sleep bouts in both the subjective day and night (Figure 8C, ANOVA: *F_3,442_* = 98.99, *p* < 0.0001), although the sleep bout length was significantly shorter than flies housed in LD, resulting in the decrease in total sleep (Figure 8D, ANOVA: *F_3,442_* = 78.45, *p* < 0.0001). Thus, it is possible that alterations in sleep in LL flies, in addition to the disruption of the circadian clock, caused the changes in alcohol toxicity. To test the role of sleep, we first determined the degree to which THIP exposure increased sleep in flies housed in LL. As expected, flies housed with THIP-containing food in constant light slept significantly more than flies on regular *Drosophila* food in LL (Figure 8E–H, mean sleep time per day: *t*_(233)_ = 29.43, *p* < 0.0001; mean sleep time, day vs. night, ANOVA: *F_3,463_* = 437.1, *p* < 0.0001; number of sleep bouts, ANOVA: *F_3,463_* = 358.1, *p* < 0.0001; mean sleep bout length, ANOVA: *F_3,463_* = 277.6, *p* < 0.0001). To separate the role of sleep from circadian regulation in mediating alcohol toxicity following a repeat binge-like alcohol exposure, we increased sleep in flies in LL as they remained under conditions of circadian disruption. We used a repeated binge-like alcohol paradigm to achieve significant levels of mortality so that potential reductions in mortality due to THIP pre-treatment could be measured. LL 10 d flies were maintained on medium containing THIP for 48 h prior to the first of three exposures to 40% alcohol vapor and then switched to normal media (Figure 7I). LL flies with THIP pre-treatment and increased sleep had significantly lower mortality than those exposed to alcohol vapor alone (Figure 8J, ANOVA: *F_3,36_* = 132.6, *p* < 0.0001). These results suggest that increased sleep was sufficient to ameliorate mortality following repeated binge-like alcohol exposure, even under conditions of circadian disruption.

### 2.7. Increasing Sleep Buffers Age-Related Susceptibility to Alcohol-Induced Mortality

**Figure 9:**
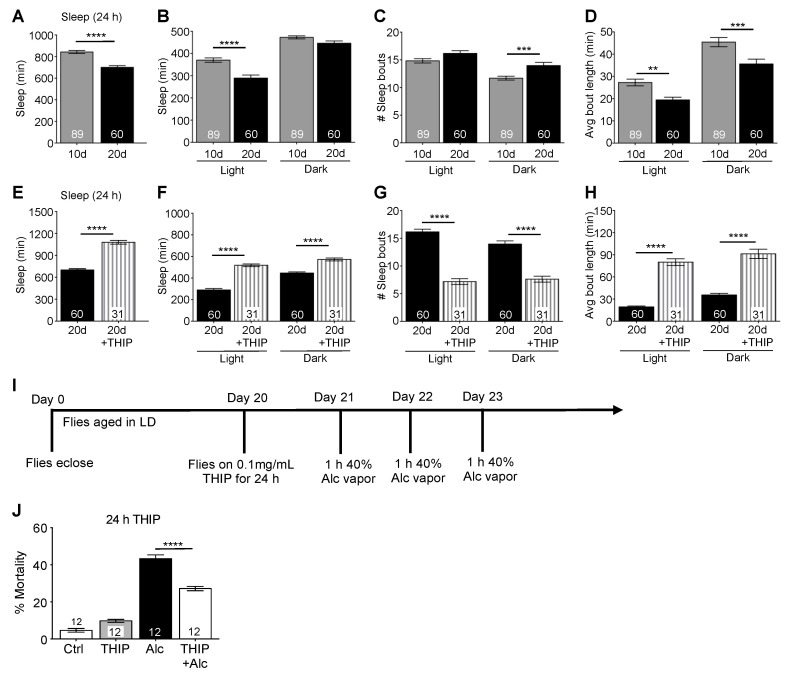
Pharmacologically increasing sleep in aging ameliorates alcohol-induced mortality. Compared to young 10 d flies, male 20 d flies grown in LD cycles exhibited significantly decreased average sleep time per day (**A**), decreased sleep during both the light and dark cycles (**B**), an increased number of sleep bouts (**C**), and decreased bout duration (**D**). (**E**–**H**) Flies 20 days of age fed 0.1 mg/mL THIP exhibited significantly greater total sleep (**E**) compared to 20 d flies on control media, (**F**) increased daytime and nighttime sleep, (**G**) a decreased number of sleep bouts, and (**H**) increased bout length. (**I**) Flies were grown in 12 h light: 12 h dark cycles and transferred to media containing 0.1 mg/mL THIP on day 19 for 24 h. On days 20, 21, and 22, flies were subjected to a 3-exposure repeated binge-like alcohol paradigm with 1 h alcohol exposure (40% alcohol vapor) occurring at ZT 9, and mortality was assessed. (**J**) Increased rest in 20 d THIP-fed flies significantly ameliorated alcohol-induced mortality compared to 20 d flies fed control media (ANOVA *F*_3,44_ = 243.8, *p* < 0.0001). * indicates significant differences between groups as calculated by Bonferroni post hoc analysis; ** *p* < 0.01, *** *p* < 0.001, **** *p* < 0.0001.

Aging is accompanied by a breakdown in circadian rhythmicity at the cellular, metabolic, and physiological levels, as well as disruptions in sleep architecture [69,88,89,90,91,92]. In recent years, chronic and binge alcohol consumption in middle-aged and older adults has significantly increased [93,94] with more than 75% of alcohol-induced poisoning deaths occurring in these age groups [11,14]. More than 10% of older adults engage in binge-drinking behavior [93]. Given that the aging population is expected to double by 2050 [95], it is necessary to identify ways to treat or ameliorate alcohol toxicity in middle-aged and older individuals. In a previous study, we showed that aging exacerbated alcohol sensitivity and mortality [69]. Middle-aged flies (20 d) exhibit shorter sleep times compared to younger flies [40] (Figure 9A, *t*_(147)_ = 6.54, *p* < 0.0001; Figure 9B, ANOVA: *F_3,294_* = 38.12, *p* < 0.0001). While we found no differences in total sleep amount during the night between 10 and 20 d flies, 20 d flies had a significantly greater number of sleep bouts with shorter duration, reflecting decreases in sleep consolidation (Figure 9C, ANOVA: *F_3,294_* = 19.21, *p* < 0.0001; Figure 9D, ANOVA: *F_3,294_* = 38.12, *p* < 0.0001). We pharmacologically induced sleep in 20 d CS flies by housing them on 0.1 mg/mL THIP for 24 h, after which they were given 1 h of alcohol exposure for three consecutive days. THIP-fed 20 d flies slept significantly more than control 20 d flies (Figure 9E–H: avg total sleep/day: *t*_(89)_ = 13.05, *p* < 0.0001; avg sleep time, day vs. night, ANOVA: *F_3,178_* = 91.72, *p* < 0.0001; number of sleep bouts, ANOVA: *F_3,178_* = 60.24, *p* < 0.0001; sleep bout length, ANOVA: *F_3,178_* = 113.0, *p* < 0.0001). Middle-aged flies housed on THIP-containing food prior to repeated binge-like alcohol exposures had significantly lower rates of mortality than those exposed to alcohol alone (Figure 9J, ANOVA: *F_3,44_* = 243.8, *p* < 0.0001). The decreased mortality observed in THIP-fed flies was not due to increased alcohol tolerance from THIP interactions, as 20 d flies given THIP for 1 h at ZT 7 followed by alcohol exposure at ZT 9 showed mortality rates similar to 20 d flies exposed to alcohol alone (Figure 9K, ANOVA: *F_3,30_* = 23.09, *p* < 0.0001). These results suggest that increased sleep was sufficient to ameliorate mortality following repeated alcohol exposures in middle-aged flies that had both circadian and sleep disruption.

### 2.8. Sleep Deprivation Inhibits Long-Term but Not Short-Term Tolerance

**Figure 10:**
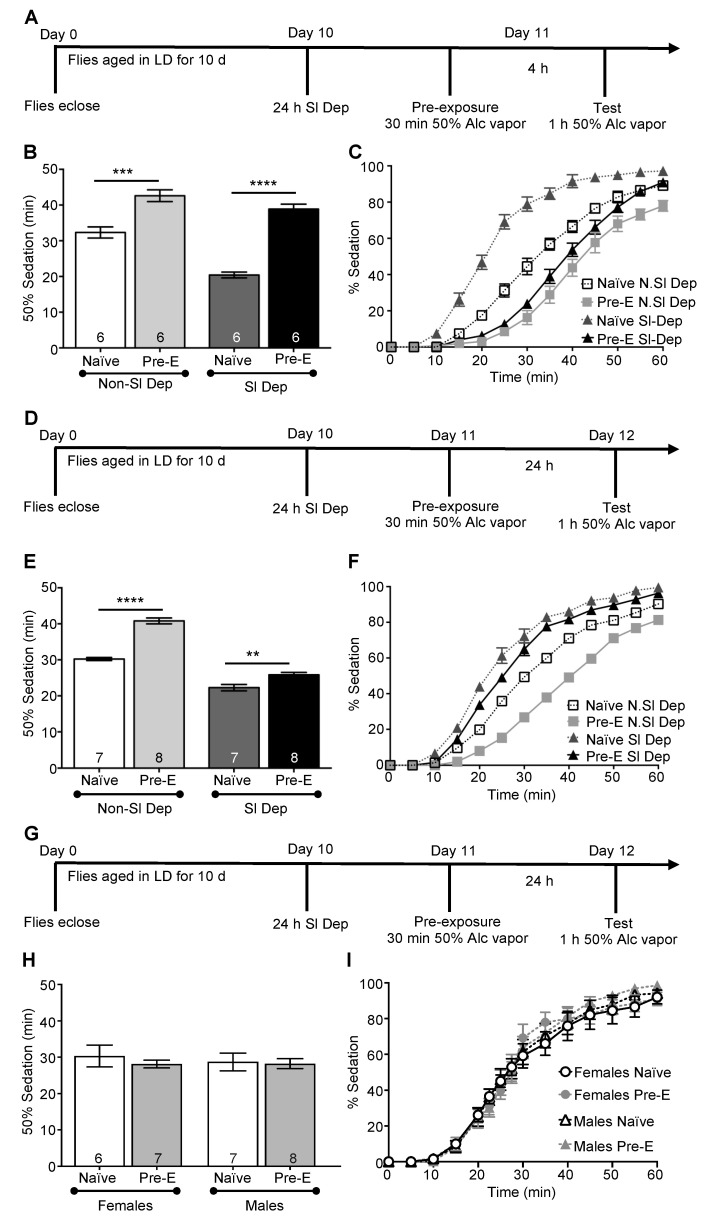
Sleep deprivation differentially affects short-term and long-term functional tolerance. (**A**–**C**) Effect of 24 h of sleep deprivation on short-term functional alcohol tolerance. (**A**) Wild-type CS flies were aged in 12:12 h LD cycle and sleep-deprived for 24 h on day 10. On day 11, flies were exposed to 50% alcohol vapor for 30 min and tested 4 h later by exposure to 50% alcohol vapor for 1 h with sedation measured. (**B**) Sleep deprivation for 24 h had no significant effect on the development of short-term acute alcohol tolerance. Both sleep-deprived and non-sleep-deprived flies exhibited alcohol tolerance (ANOVA: *F*_3,20_ = 49.62, *p* < 0.0001). (**C**) Complete time course of alcohol exposure showing percent of flies exhibiting sedation for 10 d old sleep-deprived and non-sleep-deprived flies. (**D**–**F**) Effect of sleep deprivation on development of long-term functional alcohol tolerance. CS flies were aged in 12:12 h LD cycle and sleep-deprived for 24 h on day 10. On day 11, flies were exposed to 50% alcohol vapor for 30 min and tested 24 h later by exposure to 50% alcohol vapor for 1 h at ZT 9 with sedation measured. (**E**) Non-sleep-deprived flies exhibited robust alcohol tolerance (ANOVA: *F*_3,26_ = 125.7, *p* < 0.0001) with 24 h sleep deprivation significantly dampening development of long-term functional alcohol tolerance. (**F**) Complete time course of alcohol exposure showing percent of flies exhibiting sedation for 10 d old sleep-deprived and non-sleep-deprived flies. (**G**,**H**) The effect of sleep deprivation on the development of long-term alcohol tolerance in males and females was tested. (**G**) CS flies were aged in 12:12 h LD cycle with males and females in separate groups and sleep-deprived for 24 h. On day 11, flies were exposed to 50% alcohol vapor for 30 min and tested 24 h later with exposure to 50% alcohol vapor for 1 h. (**H**) Sleep deprivation abolished the development of long-term tolerance in both males and females (ANOVA: *F*_3,24_ = 0.2390, *p* = 0.8682). (**I**) Complete time course of alcohol exposure showing percent of flies exhibiting sedation in each group. * indicates significant differences between groups as calculated by Bonferroni post hoc analysis; ** *p* < 0.01, *** *p* < 0.001, **** *p* < 0.0001.

*Drosophila* exhibit drug tolerance of repeated alcohol exposures in which the behavioral response to subsequent exposures to alcohol is lessened, similar to those observed in rodent models and humans. At the behavioral level, functional tolerance results in a decreased sensitivity to alcohol during subsequent exposures, with increased alcohol concentrations or longer alcohol exposures necessary to induce sedation [33,96,97]. In flies, rapid tolerance develops after a single alcohol exposure and can be observed during a second alcohol exposure 4 h or 24 h later [50,98]. The development of functional alcohol tolerance is dependent upon changes in neural plasticity rather than changes in the metabolism or clearance of alcohol [50,53,54,55,98,99]. Changes in neural plasticity associated with drug and alcohol tolerance share features in common with the synaptic plasticity observed in learning and memory [100,101,102]. Potentially, sleep loss affects the development of alcohol tolerance, as sleep deprivation disturbs memory formation, as seen across invertebrate and vertebrate species [103,104,105]. To investigate the effect of sleep deprivation on tolerance formed after a single alcohol exposure, 10 d wild-type flies were sleep-deprived for 24 h (ZT 3.5–ZT 3.5) and given a pre-exposure of 50% alcohol vapor for 30 min (ZT 4.5; Figure 10A). Pre-exposed sleep-deprived flies and sleep-deprived naïve flies were exposed to alcohol 4 h later at ZT 9, during which sedation was measured (Figure 10A). Similarly, non-sleep-deprived flies were pre-exposed to alcohol, with responses compared during a second alcohol exposure to naïve flies. Non-sleep-deprived flies demonstrated a robust 4 h alcohol tolerance, with significant increases observed in the time necessary for 50% of the flies to reach sedation compared to naïve flies (Figure 10B, ANOVA: *F_3,20_* = 49.62, *p* < 0.0001; Figure 10C). Surprisingly, sleep-deprived flies also demonstrated a robust 4 h alcohol tolerance (Figure 10B,C), suggesting that sleep disruption did not affect the cellular-signaling mechanisms necessary for the formation of 4 h tolerance.

To determine the effect of sleep deprivation on the formation of long-term alcohol tolerance, flies were sleep-deprived for 24 h (ZT 7.5–ZT 7.5), given a pre-exposure of 50% alcohol vapor for 30 min (ZT 8.5), and tested 24 h later at ZT 9 (Figure 10D). Groups of non-sleep-deprived flies were handled concurrently. When flies were tested 24 h after the initial alcohol exposure, sleep-deprived flies demonstrated significantly less tolerance to alcohol with a time to sedation similar to sleep-deprived naïve flies, while non-sleep-deprived flies demonstrated a robust long-term tolerance with response times significantly different than naïve flies (Figure 10E, ANOVA: *F_3,26_* = 125.7, *p* < 0.0001; Figure 10F). Although 24 h of acute sleep deprivation eliminated the sex-specific differences in alcohol sedation, it is possible that sex-specific differences occurred in the development or magnitude of functional alcohol tolerance. After mating for 72–96 h, we investigated the effects of sleep deprivation on 24 h tolerance in males and females maintained separately. We found that acute sleep deprivation inhibited long-term alcohol tolerance in both male and females, with no differences observed between pre-exposed and naïve flies (Figure 10H,I).

Although our previous research found that tolerance was not modulated by the circadian clock, we verified the effect on long-term tolerance by exposing flies to alcohol at ZT 4.5, the same time that we observed the formation of 4 h tolerance in sleep-deprived flies (Supplemental Figure S5A). Sleep-deprived flies pre-exposed to alcohol at ZT 4.5 and then subsequently exposed to alcohol at ZT 9 the following day also exhibited little or no alcohol tolerance, while non-sleep-deprived flies exhibited significant long-term tolerance (Supplemental Figure S5B,C, ANOVA: *F_3,20_* = 0.92, *p* = 0.4488). Flies (9–10 d old) were sleep-deprived for 24 h, and thus, acute sleep deprivation prior to alcohol exposure inhibited the expression of alcohol tolerance 24 h following the initial alcohol pre-exposure, while no effect was observed on the development of short-term tolerance expressed 4 h after the initial exposure. These results are consistent with the hypothesis that different molecular mechanisms are behind the development of short-term and long-term rapid alcohol tolerance, similar to differences in the formation of short- and long-term memory.

## 3. Materials and Methods

### 3.1. Fly Maintenance

All flies were maintained on standard cornmeal-molasses food at 25 °C and 60–70% relative humidity in 12:12 light:dark (LD) cycles. Canton S (CS) flies were used as the wild-type strain. *Insomniac* (*inc*) mutants and the background *w^1118^* line were generously provided by Dr. Nicholas Stavropoulos, New York University. Adult flies (~30–40 per vial) were transferred approximately every 3–4 days to maintain stress-free cultures. All experiments were carried out in an environmentally controlled dark room at 25 °C and 60–70% relative humidity under dim red light. Zeitgeber time (ZT) 0 represented lights on, and ZT 12 corresponded with lights off. For experiments performed in constant light (LL) conditions, flies were transferred to LL on the day of eclosion.

### 3.2. Alcohol Exposure

Alcohol vapor exposure was performed as previously described [51,69,106]. Four tubes, each containing ~30 flies, received a steady flow of ethanol vapor at a pre-determined percentage. Precise alcohol percentages were achieved by mixing air bubbled through deionized water and 95% ethanol (Koptec, Decon Labs, Inc. King of Prussia, PA, USA). Air flow rates were monitored throughout the experiment to ensure consistency of alcohol concentration. Water vapor controls were run simultaneously with 100% water vapor. Alcohol exposures were performed from ZT 8 to ZT 10 to avoid circadian variation in responses unless otherwise stated for a specific protocol.

### 3.3. Sleep Deprivation

Consistent sleep deprivation was achieved using gentle mechanical stimulation on a GyroMini Nutating Mixer (Labnet International, Inc. Edison, NJ, USA). Vials containing 30–50 flies were placed at an angled position from the vertical center in a larger beaker with a raised block at a fixed position protruding inside the beaker on the mini gyrator. Mixer rotation caused the vials to rotate within the beaker and then gently jump over the raised block, providing the flies with a startle movement every 2.5 s. The constant motion of the vials combined with the startle ensured consistent sleep deprivation, with no apparent injuries or increased mortality observed after 24 h of sleep deprivation. Sleep deprivation was performed in an incubator under 25 °C, 60–70% relative humidity, and 12:12 LD conditions. Non-sleep-deprived controls were housed in the same incubator.

### 3.4. Sedation

Alcohol-induced sedation was performed as previously described [48]. Briefly, flies were exposed to 50% alcohol vapor for one hour with observations of behavioral state made every five minutes following a gentle tap of the vial. Flies were scored as sedated when immobile and lacking coordinated leg movements except for spontaneous twitching [52]. The mean time to 50% sedation was calculated using a linear extrapolation.

### 3.5. Tolerance

Tolerance was determined as previously described [51]. Flies received a pre-exposure of 50% alcohol for 30 min at ZT 4.5 following a one-hour dark room acclimation period. Sedation was assessed during the pre-exposure. Flies were then returned to food vials to allow time for recovery and complete metabolism of the alcohol before testing. Testing occurred 4 h later at ZT 9 for short-term rapid tolerance. For 24 h tolerance, flies were sleep-deprived between ZT 8 and ZT 8 and then exposed to alcohol at ZT 9. Testing occurred 24 h later for long-term rapid tolerance, with all experimental groups within the experiment represented at each test. Tolerance was defined as an increase in average time to reach 50% sedation from the pre-exposure with the difference in sedation time between naïve and pre-exposed flies used for quantification.

### 3.6. Mortality

Following each alcohol exposure, flies were returned to food vials placed horizontally for approximately 2 h to allow recovery of postural control. Immediate mortality was assessed 24 h following the last alcohol exposure and then daily for 6 days. Delayed mortality referred to the cumulative mortality within seven days of the final alcohol exposure. For some experiments, a repetitive alcohol exposure protocol was used to assess alcohol-induced mortality as described previously [69].

### 3.7. Gaboxadol Treatment

Sleep was pharmacologically increased with the GABA-A agonist, 4,5,6,7-tetrahydroisoxazolo [5,4-c]pyridin-3-ol (THIP or Gaboxadol). Flies that were 10 d old (*Inc* mutants, *Dilp2* mutants, wild-type in constant light) or 20 d old (in LD) flies were transferred to Gaboxadol-containing food (0.1 mg/mL) for either 24 h or 48 h depending on the experiment prior to alcohol exposure. Flies were transferred back to non-THIP-containing media immediately prior to the 1 h habituation that preceded alcohol exposure.

### 3.8. Alcohol Absorbance

Following 24 h of sleep deprivation, batches of 20 flies were exposed to 50% alcohol vapor for 30 min at ZT 9, after which they were frozen at 0, 0.5, 1, 2, or 4 h following alcohol exposure. Alcohol absorbance was measured using an enzymatic alcohol dehydrogenase assay (ADH-NAD kit; Sigma-Aldrich, Burlington, MA, USA) per the manufacturer’s directions and as described previously [49,51]. Briefly, flies were homogenized in 200 uL refrigerated Tris-HCl (pH 7.5) buffer. Homogenate was spun at 15,000× *g* for 20 min at 4 °C. An amount of 250 uL NAD-ADH reagent was added to a 5 μL aliquot of supernatant. Absorbance was measured at 340 nm within 20 min using a 96-well plate format and a Versa-Max plate reader (Molecular Devices). Alcohol absorbance was normalized to total protein to eliminate the effect of body size variation between batches of flies.

### 3.9. Locomotor Activity Rhythms

Sleep activity was monitored using *Drosophila* activity monitors (Trikinetics, Waltham, MA, USA) as described previously [82]. To test the effectiveness of group sleep deprivation, following 24 h of sleep deprivation ending at ZT 8, flies were transferred to individual Trikinetics activity tubes with control media (1.20% agar and 5% sucrose), and sleep was measured compared to non-sleep-deprived flies transferred from group housing vials in the same manner at the same time. Similar procedures were followed for flies entrained to LL at 25 °C and for flies exposed to THIP-infused media. Sleep activity was recorded for 4 to 5 days, with data analyzed using either ClockLab or Shiny R-DAM.

### 3.10. Statistics

Statistics were performed using GraphPad Prism Version 6.0. Experimental groups were compared using analysis of variance (ANOVA). Post hoc analyses in multiple comparisons were performed using the Bonferroni correction.

## 4. Discussion

Research from our lab and others has suggested a bidirectional relationship between circadian clock dysfunction and the onset and severity of alcohol-related pathologies [18,48,51,107,108]. Social jet lag, or large shifts in sleep timing between the weekday and the weekend, is observed in numerous populations, including individuals on shift and rotating schedules [109,110], and is strongly correlated with increased alcohol use [111,112]. Due to long working hours, rotating schedules, and work-associated stress, many individuals report using alcohol as a sleep aid [61,62,113,114], which can eventually lead to an increased number of binge-drinking episodes and other detrimental effects associated with alcohol abuse [61,62,64,115]. Previous studies from our lab found that the circadian clock modulated alcohol sensitivity and toxicity and that circadian dysfunction significantly increased behavioral sensitivity to alcohol and mortality following acute and repeated alcohol exposures [48,51]. In humans, differences in individual chronotype also appear to modulate alcohol use and its associated pathologies. Individuals expressing an “evening chronotype” report significantly increased alcohol use [116,117,118,119,120,121,122,123,124,125,126,127,128]. Interestingly, individuals with an evening chronotype also have lower quality of sleep and increased daytime fatigue [129,130]. However, it is difficult to detangle the effects of circadian dysfunction from the effects of altered sleep on alcohol use.

Sleep disorders and sleep disturbances have become increasingly prevalent in modern society with longer working hours, irregular work schedules, and the prevalence of electronics, affecting more than 35% of adults and 70% of teenagers [27,73,131,132,133,134]. Insufficient sleep exacerbates the risk of developing chronic diseases and health problems, including cancer, diabetes, and neurodegenerative and psychiatric disorders [135,136,137,138,139]. Consequently, we investigated the effects of sleep loss on alcohol sensitivity and toxicity using *Drosophila melanogaster* to dissect the interactions between sleep deprivation and alcohol sensitivity and mortality. For this research, it was important to adopt a group sleep deprivation approach, as animals across phylogeny in social isolation demonstrate behavioral alterations and changes in synaptic structure and number [140]. In *Drosophila*, social isolation has been found to decrease synapse number in PDF neurons and reduce alcohol sensitivity [67,68]. Studies using rodent models have shown that social isolation increases alcohol preferences [141,142,143,144]. Therefore, we used a group-housed sleep deprivation method that significantly increased homeostatic sleep pressures in both male and females, as seen by increased sleep in the 36 h following the period of sleep deprivation.

We found that group-housed flies with acute (24 h) sleep deprivation had significantly increased sensitivity and mortality following a single binge-like exposure to alcohol. Moreover, 24 h of sleep deprivation eliminated the differential alcohol responses in sensitivity normally observed between males and females. Increased mortality following alcohol exposure occurred primarily within 24 h following alcohol exposure, with similar results observed between males and females. These effects were independent of stress or injury, as 72 h of recovery sleep prior to alcohol returned alcohol-induced behavioral responses to baseline levels with no differences seen between sleep-deprived and non-sleep-deprived flies. The increases in sensitivity and mortality were also independent of changes in metabolic tolerance, as there were no differences between sleep-deprived and non-sleep-deprived flies in alcohol absorbance or clearance. It should be noted that these experiments were all performed at approximately the same time of day (ZT 8–ZT 10), as the circadian clock regulates both alcohol-induced sedation and mortality in flies [51,69]. As ZT 9 appeared to be the time at which flies were least sensitive to alcohol, it is possible that the magnitude of the effects of sleep deprivation may differ at other times of the day or night. However, given the robustness of the effects of sleep deprivation, it is likely that sleep deprivation overrides the circadian modulation of alcohol response, as was seen with male and female differences. Thus, sleep deprivation changed both immediate alcohol sensitivity and acute alcohol toxicity after a single binge-like alcohol exposure. Our data highlighted the phylogenetic conservation across species, showing a correlation between sleep loss and alcohol behaviors.

Studies from rodents and humans outline a correlation between sleep loss and increased severity of alcohol behavioral responses, including increased alcohol intake, accelerated development of alcohol abuse, dependence, and relapse following alcohol abstinence [145,146,147,148]. In mice, alcohol dose-dependently increased hyperactive locomotor activity in open-field tests, with acute sleep deprivation for 48 h abolishing these stimulatory effects [149]. Insufficient sleep (<8 h per night) is correlated with an increased number of drinking sessions in adolescents and young adults [150,151,152]. College-aged students are considered a vulnerable population for risk-taking behaviors, and multiple studies have shown a strong correlation between poor sleep quality and excessive alcohol intake and the accompanying consequences for mental health and academic performance, including increased rates of depression, anxiety, and psychological stress, as well as academic issues in these students [63,153,154]. Insufficient and poor-quality sleep also appear to predict the onset of alcohol abuse and its adverse consequences [65,155,156,157,158]. Sleep disturbances observed in children 3–5 years of age predicted the early onset of alcohol use at ages 12–14 [159]. This is particularly harmful because recovering alcoholics who use alcohol as a sleep aid are three times more likely to relapse in 12 months [22,23,160]. Altogether, these studies emphasize disturbed sleep as a potent risk factor for the initiation of alcohol use, the escalation of problems associated with alcohol abuse, and the hindrance of recovery from alcohol-use disorders.

With the genetic tools and mutants available, *Drosophila* provided a suitable model system to test the relationship between chronic sleep disturbances and alcohol-induced pathologies. Using flies with mutations in the *insomniac* gene (*inc*) that provided a model mirroring chronic sleep restriction, we found that *inc* mutants had significantly increased mortality following alcohol exposure than background controls. *Inc* mutant flies were surprisingly less sensitive to the sedative effects of alcohol compared to their background controls, supporting previous research that the different physiological consequences of alcohol can be regulated separately. Although the mechanism through which sleep buffers alcohol toxicity is unknown, it is possible that the changes in oxidative stress in the *inc* mutant flies may contribute to the change in alcohol toxicity. *Inc^1^* and *inc^2^* mutant flies have a reduced lifespan compared to genetic background controls, with *inc^1^* flies having a maximum lifespan of approximately 50 days, *inc^2^* mutants with an approximate 60-day maximum, and wild-type flies with an approximate 70-day maximum lifespan [75]. The *inc* gene seems necessary for mediating the oxidative stress response, as reducing *inc* both globally and neuronally significantly increases mortality following a single injection to the paraquat, a common inducer of oxidative stress [75,161]. Support for this hypothesis is found in previous research demonstrating that pharmacologically increasing sleep in *inc* mutant flies using gaboxadol significantly decreased the sensitivity to paraquat-induced oxidative stress [161]. Sleep loss was also shown to increase reactive oxygen species in the gut [154], raising the possibility that peripheral mechanisms also contribute to increased alcohol toxicity. As changes in sleep potentially impact multiple physiological processes in the central nervous system, as well as in peripheral organs, the precise mechanism through which sleep buffers alcohol toxicity is undoubtedly the focus of future studies.

To independently examine the effects of short sleep on alcohol mortality, we used another short-sleep mutant that maintained a functional circadian clock, *Dilp2*. Although *Dilp2* mutants exhibit slight developmental delays, these fly mutants are long-lived, with between an 8 and 13% increase in lifespan observed in both male and female flies [77]. The insulin-like peptides, *Dilp2*, *3*, and *5* are expressed in the median neurosecretory cells of the adult fly brain [77,162]. In *Dilp2* mutant flies, *Dilp3* and *Dilp5* are upregulated with a large degree of compensation apparent [77]. The role of insulin signaling in alcohol responses was previously investigated, reporting that flies with reduced levels of the insulin receptor demonstrated an increased sensitivity to alcohol [163]. However, we found that *Dilp2* mutants had no significant difference in the rate or sensitivity to alcohol-induced sedation, probably due to compensation from other insulin-like peptides in *Drosophila*. *Dilp2* mutants had significantly higher levels of alcohol-induced mortality compared to background controls. Similar to the *inc* mutants, we found that increasing sleep in *Dilp2* flies using gaboxadol significantly mitigated alcohol-induced mortality. Previously, researchers found that overexpression of *Dilp5* or *Dilp6* ameliorated developmental alcohol toxicity in larvae, whereas overexpression of *Dilp2* had no effect on alcohol toxicity unless it was ectopically expressed throughout the animal [164].

Pharmacologically increasing sleep in flies maintained in LL with dysfunctional circadian clocks and middle-aged wild-type flies was sufficient to significantly reduce alcohol-induced mortality. Gaboxadol increased the total sleep duration, as well as significantly increasing sleep bout length, suggesting a greater consolidation of sleep. Both increased total sleep and increased sleep consolidation suggest that improved sleep quality could aid in mitigating alcohol-induced pathologies. Although there have been few studies examining the relationship between sleep health and alcohol toxicity, sleep loss and decreased sleep consolidation have been shown to reduce reproductive output, accelerate aging, and increase the accumulation of reactive oxygen species and death in flies [165,166]. In humans, increasing sleep in adolescents was correlated with decreased risk of emotional and cognitive disruption, as well as lowered risk of obesity [167]. In addition, increasing sleep by 30 min for 3 days over the weekend in healthy industrial workers and individuals susceptible to obesity significantly increased insulin sensitivity and had a restorative effect of sleep on metabolic homeostasis [168,169]. Finally, increasing sleep in older adults significantly improved performance on visual tasks and stabilized memory recall [170]. Although more specific research needs to be conducted assessing the direct effects of increased sleep on alcohol toxicity in vulnerable groups, these data suggest a role for sleep as a buffer to protect against the toxic effects of alcohol in populations vulnerable to chronic sleep loss, such as aged adults and shift workers.

The development of acute tolerance to alcohol is a distinct and critical behavioral metric used to gauge propensity for alcohol dependence and abuse [171], which can be separated from alcohol sensitivity and alcohol toxicity. Similar to mammals, acute exposure to a high concentration of alcohol induces functional tolerance in *Drosophila* at the behavioral [50,98,172] and the molecular levels [99,173,174,175,176]. Functional alcohol tolerance is dependent on changes in neuronal strength and connectivity or synaptic plasticity [99,173,174]. Consistent with previous findings, we observed tolerance at 4 h and 24 h following a short pre-exposure to alcohol vapor [50,51]. We found that sleep deprivation eliminated or dampened the development of tolerance at 24 h in both male and female flies but had no effect on tolerance observed at 4 h. Potentially, acute sleep deprivation selectively impaired the cellular and molecular processes necessary for encoding long-term rapid tolerance to alcohol without severe disruption of the mechanisms necessary for the development of 4 h tolerance. In fact, previous studies have demonstrated altered expressions of rapid tolerance in flies with mutations in genes necessary for learning and memory [46,98,177]. For example, the gene *dunce* (*dnc*) encodes a phosophodiesterase required for cAMP degradation and is necessary for behavioral and synaptic plasticity [178,179]. Originally identified as a learning mutant [180,181], *dnc* mutant flies exhibit significant sleep deficits [182] and are incapable of forming rapid tolerance [183,184]. Time-dependent differences in the effects of sleep deprivation can also be seen for memory with acute sleep deprivation, affecting the consolidation of long-term but not short-term hippocampal-dependent memory in mice [185,186]. Together with support from existing research, the results from our studies suggest that sleep deprivation selectively impacted processes underlying synaptic plasticity to affect the development of long-term rapid tolerance. In conclusion, the results from our study started to dissociate the role of sleep in modulating alcohol toxicity from the regulation of alcohol neurobiology by the circadian clock. These results lay the groundwork for future studies and treatments considering sleep quality and sleep duration as an important component of alcohol use disorder and alcohol-induced pathologies.

## Data Availability

The data underlying this article are available in the article and in its online supplementary material. Detailed data for individual samples will be shared on reasonable request to the corresponding author. An early version of this manuscript (the authors’ original version) prior to peer review may be found in bioRxiv.

## Supplementary Materials

The following supporting information can be downloaded at: https://www.mdpi.com/article/10.3390/ijms232012091/s1.
