# Supplementary material for: Sleep Modulates Alcohol Toxicity in Drosophila
Source: Int J Mol Sci. 2022 Oct 11;23(20):12091. doi: 10.3390/ijms232012091 (PMC9603330; doi:10.3390/ijms232012091)
Supplement: Supplementary file 1 [file ijms-23-12091-s001.zip › ijms-1888990-supplementary.pdf]

Supplementary Figure Legends

Figure S1: Mechanical sleep deprivation increases sleep fragmentation and rebound sleep in young flies. A-B) Comparison of sleep bouts between sleep-deprived (SLD) and non-sleep deprived (NSD) 10 d CS flies in the first 3 h following sleep deprivation. Compared to NSD flies, SLD male flies have significantly increased number of sleep bouts and decreased sleep bout duration, whereas SLD females the same number of bouts but longer bout duration than NSD females (A, ANOVA: $F_{3,108} = 7.16$, $p < 0.001$; B, ANOVA: $F_{3,108} = 27.62$, $p < 0.05$, asterisks indicate significance in post-hoc comparisons). C-D) During the first night following sleep deprivation, both female and male SLD flies exhibit greater fragmented sleep compared to their NSD controls with significantly increased number of sleep bouts (C, ANOVA: $F_{3,108} = 35.17$, $p < 0.001$) and decreased bout duration (D, ANOVA: $F_{3,108} = 27.62$, $p < 0.05$). E-G) SLD male and female flies continue to exhibit significantly increased total sleep, albeit fragmented sleep, on day 2 following sleep deprivation, with significantly longer total sleep time during the light and dark cycle (E, ANOVA: $F_{7,218} = 123.9$, $p < 0.001$) and increased number of sleep bouts during the dark cycle (F, ANOVA: $F_{7,218} = 30.48$, $p < 0.001$) although no significant changes were observed in duration of sleep bouts (G, ANOVA: $F_{7,218} = 26.79$, $p = 0.4208$). H-J) While SLD flies still show increased sleep even four-five days following sleep deprivation, total sleep levels approach that of NSD flies reflecting a return towards more normal sleep patterns with most of the increased sleep occurring in the day (H, ANOVA: $F_{7,168} = 278.1$, $p < 0.05$]. Females continue to have more sleep bouts when sleep-deprived (I, ANOVA: $F_{7,168} = 39.33$, $p < 0.05$), although bout length is shorter in SLD females at night (J, ANOVA: $F_{7,168} = 96.62$, $p < 0.001$).

Figure S2: Acute sleep deprivation increases sensitivity to alcohol-induced sedation A) 5 or 6 d old CS male and female flies were sleep deprived (SLD) for 24 h then exposed to 50% alcohol vapor for 1 h. Sensitivity to sedation was measured by counting the number of flies sedated every 5 min. B) Sleep deprivation significantly exacerbates alcohol-induced sedation in young male and female flies (ANOVA $F_{3,25} = 51.99$, $p < 0.0001$). Mean time necessary for 50% of the flies to become sedated during alcohol exposure and standard error of the mean plotted for all experiments. C) Complete time course of alcohol exposure showing percent of flies exhibiting sedation for 6 or 7 d old SLD and non-sleep deprived (NSD) male and female flies. D) Young (3 d old flies) group-housed mixed sex populations were sleep deprived for 24 h then exposed to 50% alcohol vapor for 1 h. E) SLD flies sedate faster compared to NSD flies, indicating increased sensitivity to alcohol ($t_{(6)} = 16.65$, $p < 0.001$). N shown on bars for each group is the number of vials tested for each group with ~30 flies per vial. F) Complete time course of alcohol exposure showing percent of flies exhibiting sedation for 3 d old SLD and NSD flies.

Figure S3: Acute sleep deprivation exacerbates alcohol-induced mortality in younger male and female flies following a single exposure to alcohol. A) 5 or 6 d old CS male and female flies were sleep deprived (SLD) for 24 h then exposed to 50% alcohol vapor for 1 h. Mortality was assessed 24 h following alcohol exposure. B) SLD flies exhibited a significant increase in mortality after alcohol exposure compared to NSDd flies (ANOVA $F_{3,11} = 17.64$, $p < 0.001$). N shown on bars for each group is the number of vials of flies tested for each group.

Figure S4: Assessment of THIP treatment and alcohol responses in w^{1118} flies. A) 10-day old w^{1118} flies were given 0.1mg/mL THIP and sleep deprived for 24 h, then exposed to 50% alcohol vapor for 1 h. Sensitivity to sedation was measured by counting the number of flies sedated every 5 min. B-C) NSD THIP treated w^{1118} flies exhibited similar behavioral response to alcohol-induced sedation as NSD non-fed

THIP w^{1118} flies. In contrast, SLD THIP-fed w^{1118} flies were significantly more sensitive to alcohol-induced sedation (B, ANOVA: $F_{2,23} = 15.22$, $p < 0.05$). C). Complete time course of alcohol exposure showing percent of flies exhibiting sedation for 10 d old NSD and SLD flies. D-E) Alcohol-induced mortality was also assessed. No significant differences were observed in mortality at 24 h (D, ANOVA: $F_{2,18} = 15.22$, $p = 0.7892$) and 7 d (E, ANOVA: $F_{2,11} = 1.127$, $p = 0.7892$) following acute exposure to alcohol vapor in NSD, NSD THIP-fed and SLD THIP-fed flies.

Figure S5: Sleep deprivation decreases formation of long-term functional tolerance 29 h following pre-exposure to alcohol. The effect of sleep deprivation on the development of long-term functional tolerance 29 h after a short pre-exposure to alcohol was tested. A) CS flies were aged in 12:12 h LD cycle and sleep deprived for 24 h on day 10. On day 11, flies were exposed to 50% alcohol vapor for 30 min at ZT 4.5 and tested 29 h later by exposing to 50% alcohol vapor for 1 h with sedation being measured. B) NSD flies exhibited robust alcohol tolerance, with sleep deprivation severely dampening the development of long-term functional alcohol tolerance (ANOVA $F_{3,15} = 106.0$, $p < 0.01$). C) Complete time course of alcohol exposure showing percent of flies exhibiting sedation.

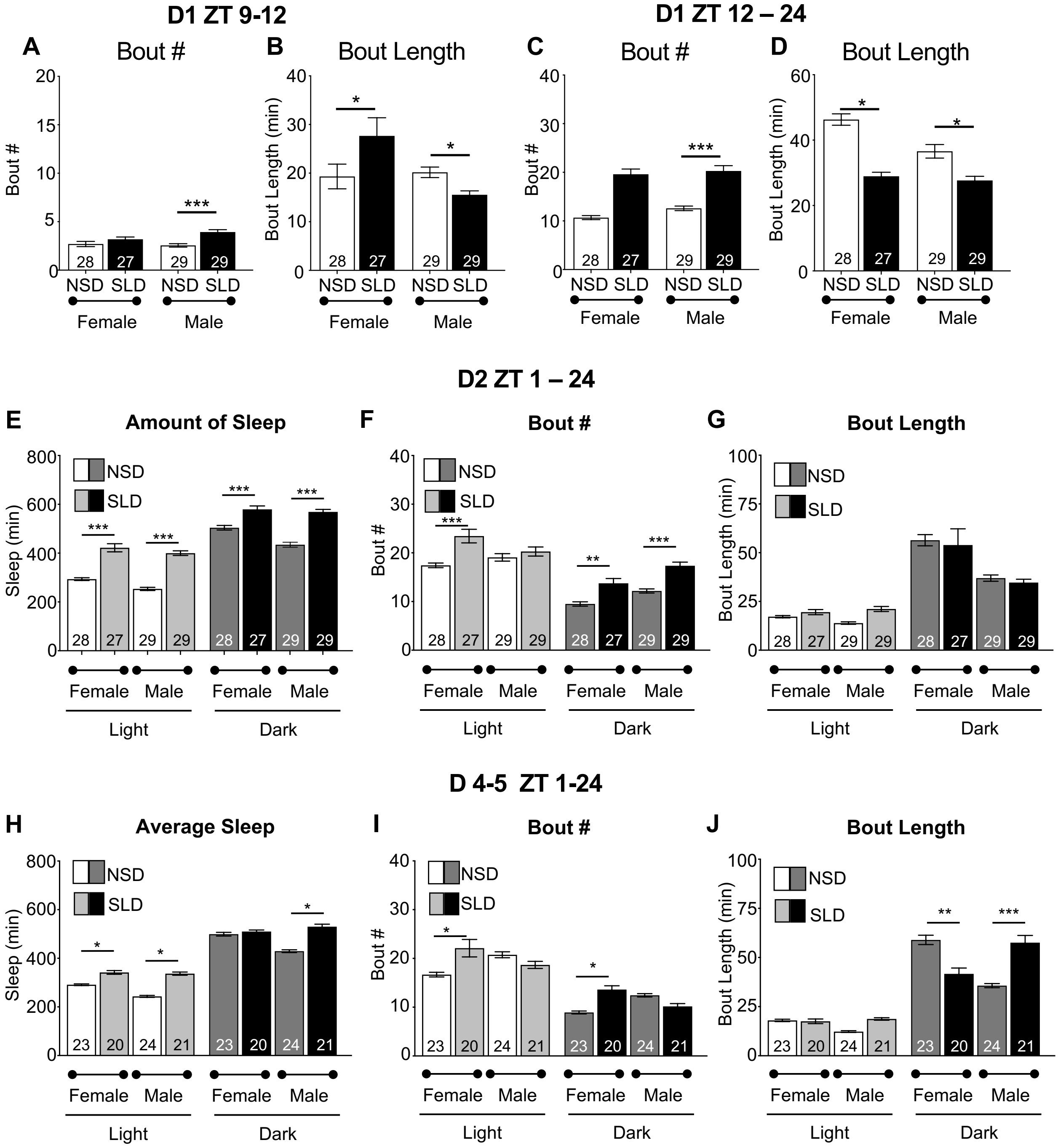
D1 ZT 9-12
D1 ZT 12 – 24
A
Bout #
B
Bout Length
C
Bout #
D
Bout Length
Bout #
Bout Length (min)
NSD SLD NSD SLD
Female Male
D2 ZT 1 – 24
E
Amount of Sleep
F
Bout #
G
Bout Length
NSD
SLD
Sleep (min)
Light
Dark
D 4-5 ZT 1-24
H
Average Sleep
I
Bout #
J
Bout Length

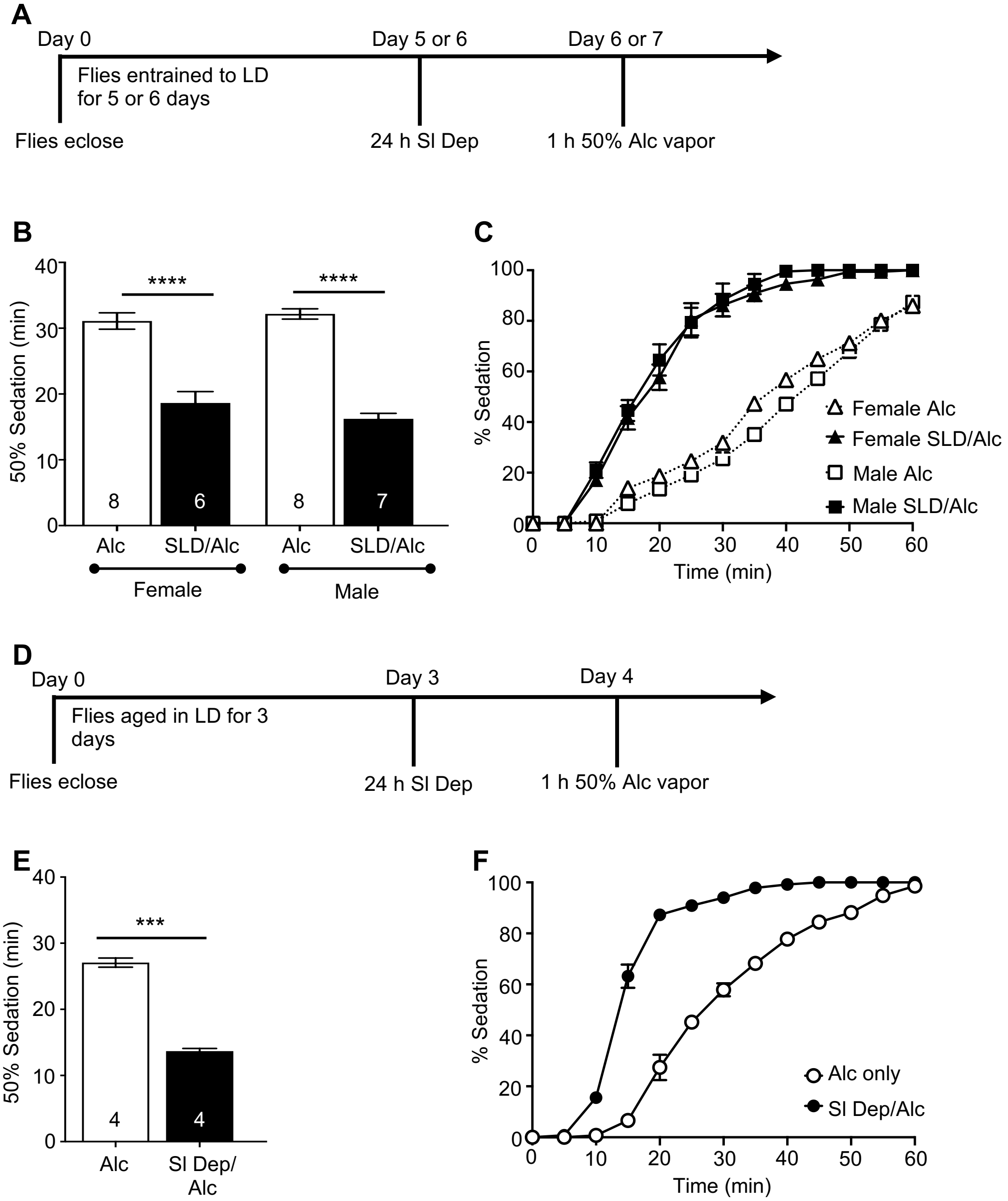
A
Day 0
Day 5 or 6
Day 6 or 7
Flies entrained to LD for 5 or 6 days
Flies eclose
24 h Sl Dep
1 h 50% Alc vapor
B
50% Sedation (min)
****
8
6
8
7
Alc
SLD/Alc
Alc
SLD/Alc
Female
Male
C
% Sedation
Time (min)
Female Alc
Female SLD/Alc
Male Alc
Male SLD/Alc
D
Day 0
Day 3
Day 4
Flies aged in LD for 3 days
Flies eclose
24 h Sl Dep
1 h 50% Alc vapor
E
50% Sedation (min)
***
4
4
Alc
Sl Dep/ Alc
F
% Sedation
Time (min)
Alc only
Sl Dep/Alc

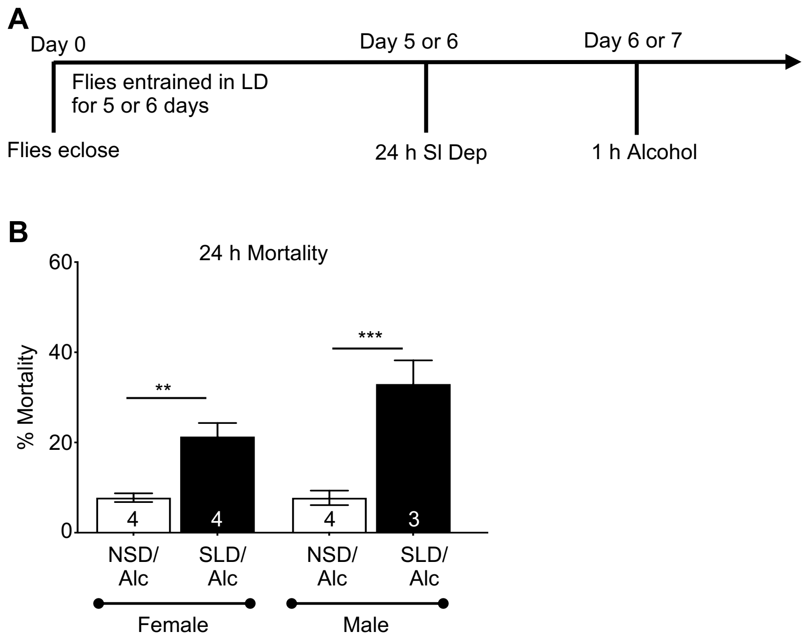

A
Day 0
Day 5 or 6
Day 6 or 7
Flies entrained in LD
for 5 or 6 days
Flies eclose
24 h Sl Dep
1 h Alcohol
B
24 h Mortality
% Mortality
60
40
20
0
**
***
4
4
4
3
NSD/
Alc
SLD/
Alc
NSD/
Alc
SLD/
Alc
Female
Male

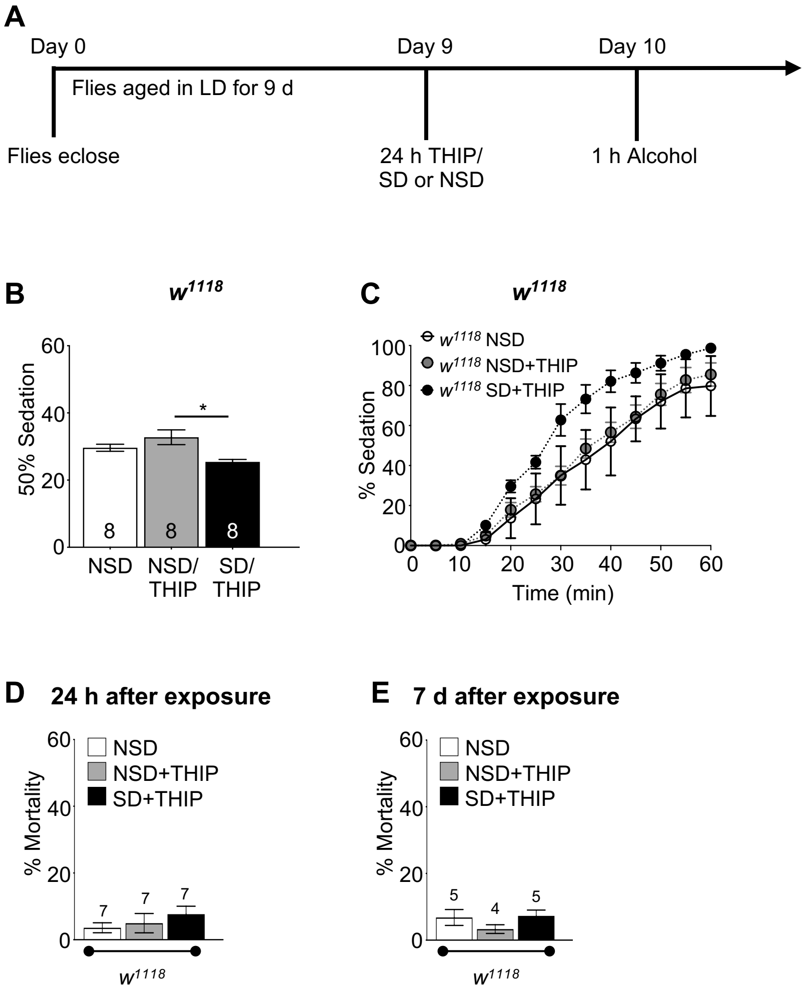

A
Day 0
Day 9
Day 10
Flies aged in LD for 9 d
Flies eclose
24 h THIP/ SD or NSD
1 h Alcohol
B
w1118
50% Sedation
60
40
20
0
*
8
8
8
NSD
NSD/ THIP
SD/ THIP
C
w1118
w1118 NSD
w1118 NSD+THIP
w1118 SD+THIP
% Sedation
100
80
60
40
20
0
0
10
20
30
40
50
60
Time (min)
D
24 h after exposure
% Mortality
60
40
20
0
NSD
NSD+THIP
SD+THIP
7
7
7
w1118
E
7 d after exposure
% Mortality
60
40
20
0
NSD
NSD+THIP
SD+THIP
5
4
5
w1118

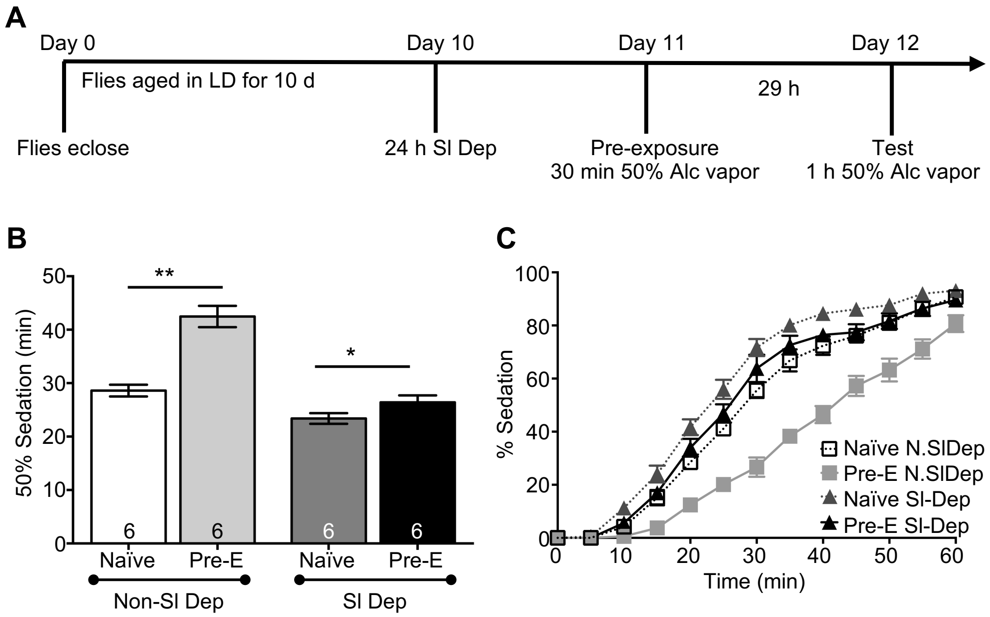
A
Day 0
Day 10
Day 11
Day 12
Flies aged in LD for 10 d
29 h
Flies eclose
24 h Sl Dep
Pre-exposure
30 min 50% Alc vapor
Test
1 h 50% Alc vapor
B
50% Sedation (min)
**
*
Naïve
Pre-E
Naïve
Pre-E
Non-Sl Dep
Sl Dep
C
% Sedation
Time (min)
Naïve N.SlDep
Pre-E N.SlDep
Naïve Sl-Dep
Pre-E Sl-Dep
